# Synthesis and crystal structures of three silicon complexes with *N*-[(2-hy­droxy­naphthalen-1-yl)methyl­idene]alanine

**DOI:** 10.1107/S2056989026008406

**Published:** 2026-08-20

**Authors:** Anke Schwarzer, Sabine Fels, Uwe Böhme

**Affiliations:** a Institut für Organische Chemie, Technische Universität Bergakademie Freiberg, Leipziger Str. 29, 09599 Freiberg, Germany; b Institut für Anorganische Chemie, Technische Universität Bergakademie Freiberg, Leipziger Str. 29, 09599 Freiberg, Germany; University of Neuchâtel, Switzerland

**Keywords:** crystal structure, Schiff base, silicon complex, chelate ligand

## Abstract

The reaction of the Schiff base ligand *N*-[(2-hy­droxy­naphthalen-1-yl)methyl­idene]alanine, H_2_*L*, with di­chloro­diorganosilicon compounds, Cl_2_Si*R*_2_, yields silicon complexes *L*Si*R*_2_. The crystal structure analyses of (*RS*)-dimethyl{*N*-[(2-oxidonaphthalen-1-yl)methyl­idene]alaninato}silicon, C_16_H_17_NO_3_Si, (**1**), (*RS*)-di­ethenyl{*N*-[(2-oxidonaphthalen-1-yl)methyl­idene]alaninato}silicon, C_18_H_17_NO_3_Si, (**2**), and (*RS*)-methyl­{*N-*[(2-oxidonaphthalen-1-yl)methyl­idene]alaninato}phenylsilicon chloroform hemisolvate, 2(C_21_H_19_NO_3_Si)·CHCl_3_, (**3**), allow a detailed analysis of the coordination geometries of the penta­coordinated silicon complexes. All three complexes adopt a distorted trigonal–bipyramidal coordination geometry. The apical positions in these coordination polyhedra are occupied by the oxygen atoms O1 and O2, whereas the equatorial positions are occupied by the nitro­gen atoms N1 and the carbon atoms of the organic groups at the silicon atom. In all three compounds, the O3 oxygen atoms inter­act in bifurcated C—H⋯O inter­actions with the imine hydrogen atoms, leading to stable zigzag chains. Differences between the three structures are found in the support of this inter­action *via* other C—H⋯O or aryl inter­actions. While **1** exhibits π–π stacking, **2** and **3** shows C—H⋯π contacts. This study demonstrates the ability of the H_2_*L* ligand to form hypercoordinate silicon complexes.

## Abstract 2

The reaction of the Schiff base ligand *N*-[(2-hy­droxy­naphthalen-1-yl)methyl­idene]alanine, H_2_*L*, with di­chloro­diorganosilicon compounds, Cl_2_Si*R*_2_, yields penta­coordinated silicon complexes LSi*R*_2_.

## 1. Chemical context

Schiff bases are versatile ligands widely used in coordination chemistry (Calligaris & Randaccio, 1987 ▸; Hernández-Molina & Mederos, 2004 ▸; Vigato & Tamburini, 2008 ▸). The presence of additional *ortho*-hy­droxy and carb­oxy groups enables the formation of polydentate ligand systems. In recent years, Schiff base complexes of silicon have attracted increasing inter­est owing to their potential medical applications (Arya *et al.*, 2024 ▸; Sharma *et al.*, 2003 ▸; Singh *et al.*, 2010 ▸, 2017 ▸). We are inter­ested in silicon complexes of Schiff base ligands from a structural point of view. Such complexes can adopt tetra­hedral (Böhme *et al.*, 2008 ▸, 2021 ▸; Böhme & Haushälter, 2009 ▸), distorted trigonal–bipyramidal (Böhme & Fels, 2013*a* ▸,*b* ▸, 2023 ▸, 2024 ▸; Schwarzer *et al.*, 2018 ▸), and octa­hedral (Mucha *et al.*, 1998 ▸, 1999 ▸) coordination geometries.
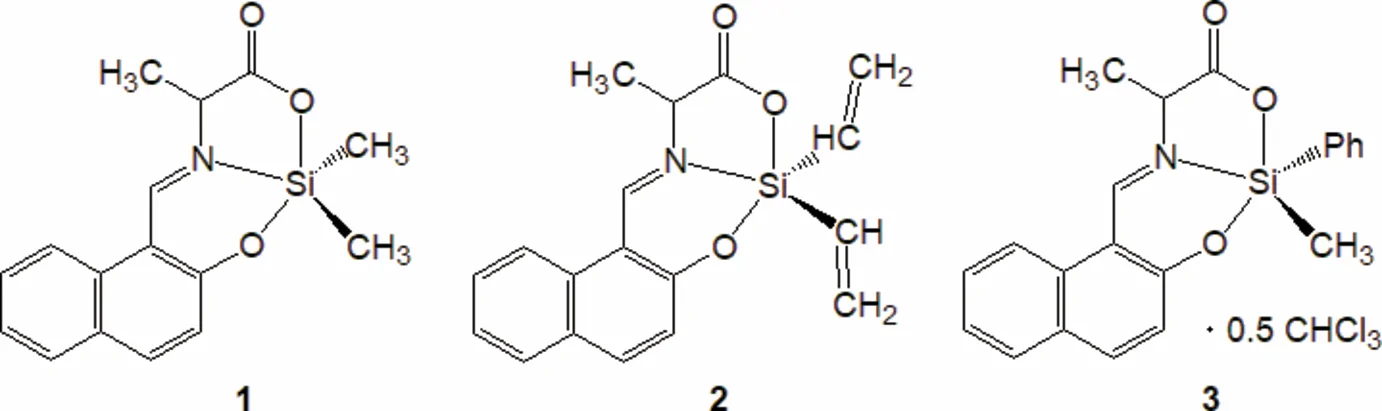


Herein, we report the synthesis, characterization and crystal structure analyses of three silicon complexes with the Schiff base ligand *N*-[(2-hy­droxy­naphthalen-1-yl)methyl­idene]alanine (H_2_*L*). The ligand is deprotonated during complex formation and coordinates as the corresponding dianion to the silicon atoms in complexes **1**–**3**. No chiral complexes were isolated, since the racemic amino acid alanine was used for the preparation of the Schiff base ligand.

## 2. Structural commentary

**Figure 1:**
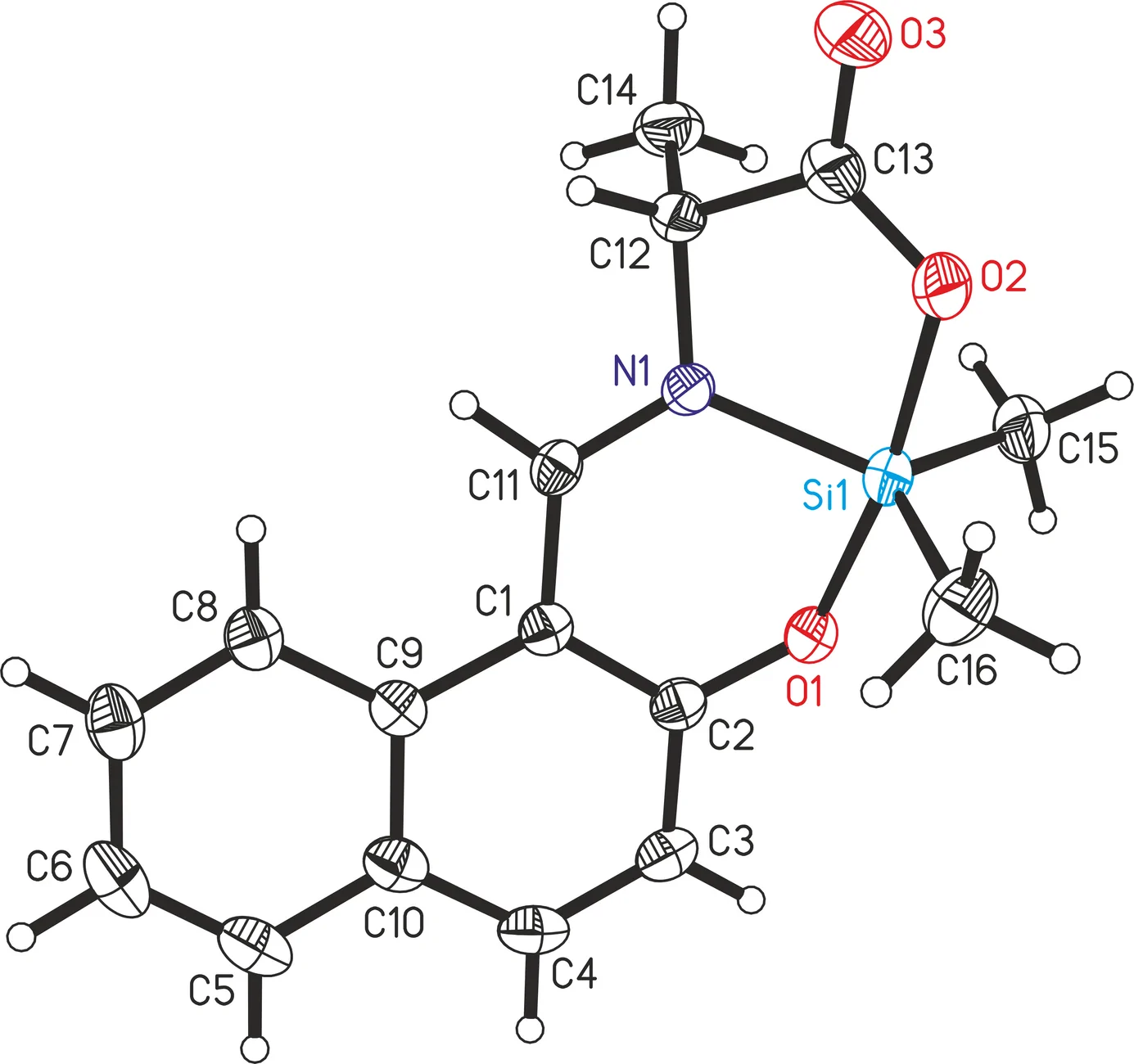
The mol­ecular structure of **1** showing the atom-labelling scheme. Atomic displacement parameters are drawn at the 50% probability level.

Compound **1** (Fig. 1 ▸) crystallizes in the monoclinic space group *P*2_1_*/n* with one mol­ecule in the asymmetric unit. The tridentate Schiff base ligand occupies three coordination sites at the silicon atom. Two methyl groups at the silicon atom complete the coordination sphere to a penta­coordinate complex. Bond lengths and angles are given in Table 1 ▸.

**Figure 2:**
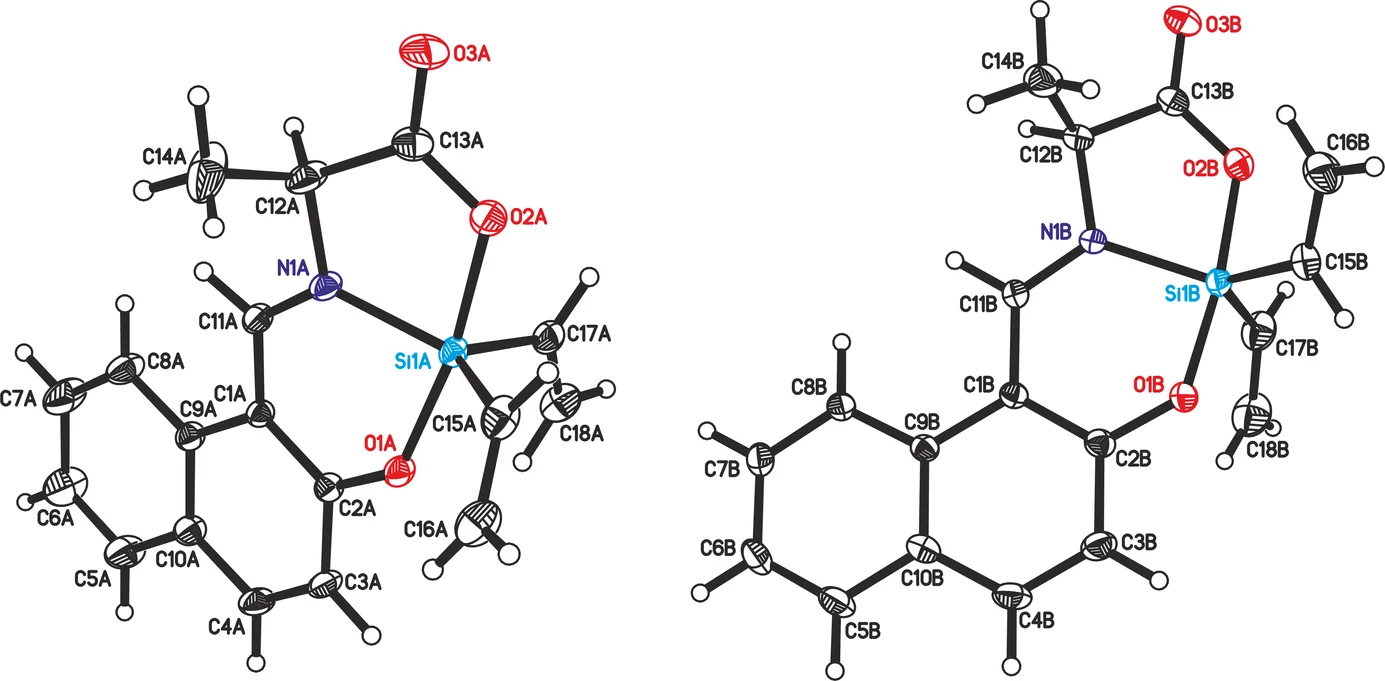
The two crystallographic independent mol­ecules of **2** showing the atom-labelling scheme. Atomic displacement parameters are drawn at the 50% probability level.

Compound **2** crystallizes in the triclinic space group *P*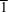 with two crystallographic independent mol­ecules in the asymmetric unit. The coordination geometry at the silicon is very similar to that in **1**. The main difference between the two crystallographically independent mol­ecules of **2** is the orientation of the vinyl groups at the silicon atom (see Fig. 2 ▸). Whereas in mol­ecule **A** the vinyl groups are directed in the same direction (‘both down’ in Fig. 2 ▸), the vinyl groups in mol­ecule **B** are directed in opposite directions (‘down and up’ in Fig. 2 ▸). Beside this obvious difference, both mol­ecules have very similar features regarding bond lengths and angles (see Table 2 ▸).

**Figure 3:**
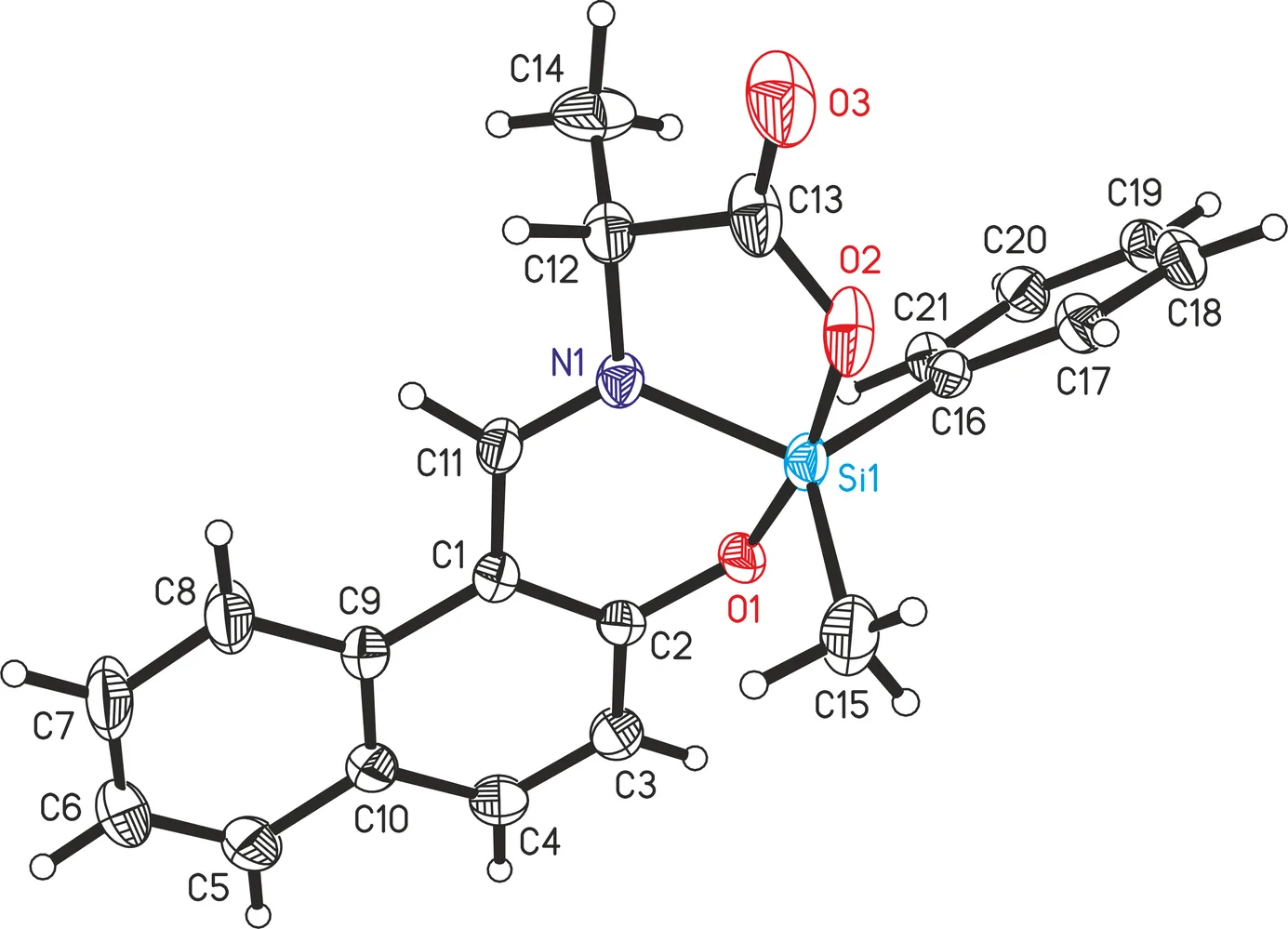
The mol­ecular structure of **3** showing the atom-labelling scheme. The chloro­form mol­ecule is omitted. Atomic displacement parameters are drawn at the 50% probability level.

**Figure 4:**
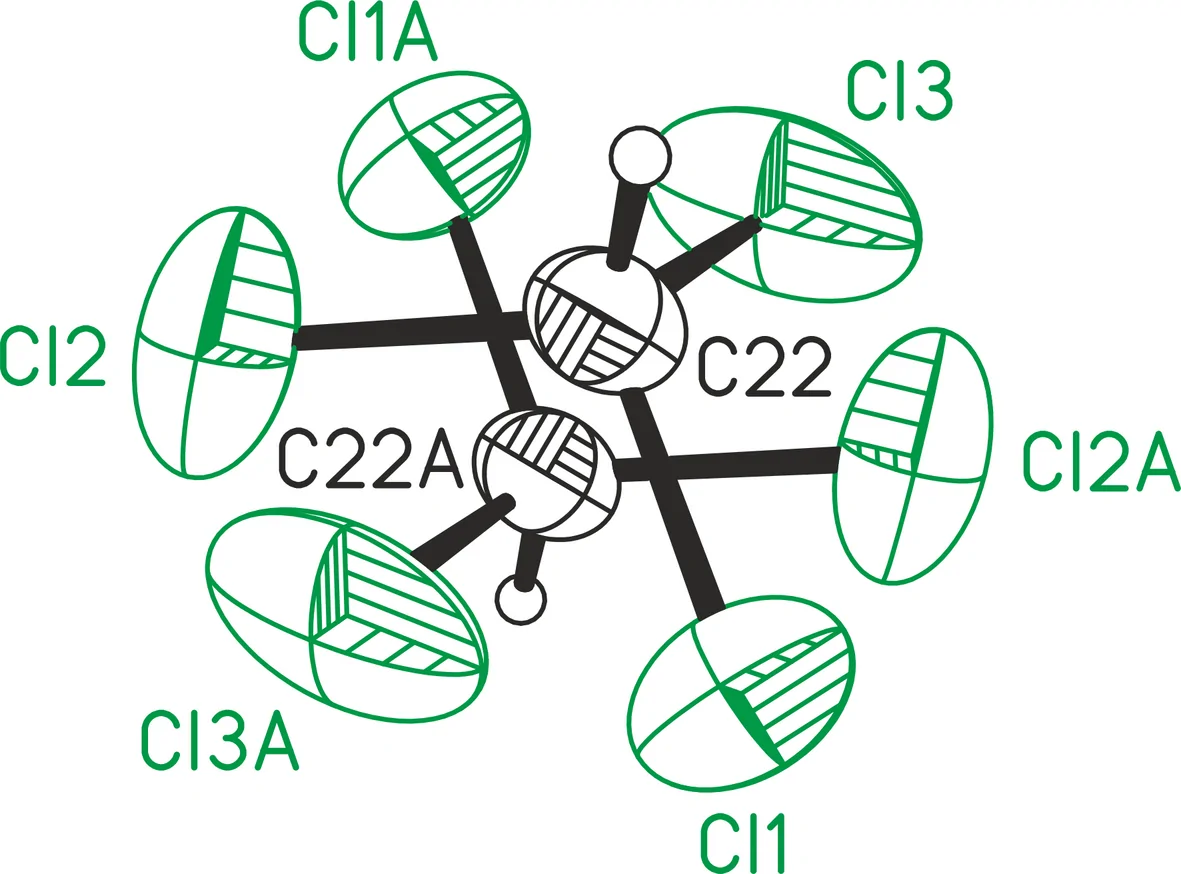
The disordered chloro­form mol­ecule in the crystal structure of **3**. Atomic displacement parameters are drawn at the 50% probability level.

Compound **3** crystallizes in the monoclinic space group *I*2_1_/*a* with one complex mol­ecule (Fig. 3 ▸) and a disordered chloro­form mol­ecule in the asymmetric unit. The chloro­form mol­ecule is disordered around a special position (see Fig. 4 ▸), which leads to a formal ratio of one silicon complex mol­ecule to half a chloro­form mol­ecule. Bond lengths and angles are given in Table 3 ▸.

The *N*-[(2-oxidonaphthalen-1-yl)methyl­idene]alaninate ligand has a planar naphthyl unit in all three complexes. The imine bond (C11—N1), with values ranging from 1.309 (2) Å in compound **3** to 1.313 (2) Å in mol­ecule **A** of **2**, is significantly shorter than a typical C—N single bond [*ca*. 1.47 Å, based on the sum of Pauling covalent radii for C (0.77 Å) and N (0.70 Å)], indicating pronounced double-bond character (Pauling, 1962 ▸). The slight elongation relative to an idealized, fully localized imine bond (∼1.28 Å) suggests partial π-electron delocalization involving the naphthyl group. The Si—O1 bonds are shorter than the Si—O2 bonds. This difference can be explained with the stronger electronegative character of the naphthyl-bound oxygen atoms O1 in comparison to the carboxyl-type atoms O2. The Si1—N1 and Si1—C bonds have similar lengths as in comparable penta­coordinate silicon complexes (Böhme & Fels, 2013*a* ▸, 2023 ▸, 2024 ▸; Schwarzer *et al.*, 2018 ▸).

The coordination geometry of the penta­coordinated silicon atoms in compounds **1**–**3** was analysed using the Addison parameter, τ (Addison *et al.*, 1984 ▸). A value of τ = 0 corresponds to a perfect square pyramid, whereas τ = 1 corresponds to a perfect trigonal bipyramid. The parameter is calculated as τ = (β – α)/60°, where β and α are the largest and second-largest angles at the central atom, respectively. The parameters are τ = 0.84 (**1**), 0.70 (mol­ecule **A** of **2**), 0.84 (mol­ecule **B** of **2**), and 0.84 (**3**), that means all three complexes are distorted trigonal bipyramids. The apical positions in these coordination polyhedra are occupied by the oxygen atoms O1 and O2, whereas the equatorial positions are occupied by the nitro­gen atoms N1 and the carbon atoms of the organic groups at the silicon atom. The strongest distortion of the coordination geometry is observed in mol­ecule **A** of compound **2**. This is caused by the ‘both down’ conformation of the vinyl groups, which leads to a larger N1*A*—Si1*A*—C15*A* angle of 127.55 (7)°. Furthermore, each vinyl group inter­acts in intra­molecular C—H⋯O contacts, while in mol­ecule **2A**, both vinyl groups inter­act with O1*A* and in mol­ecule **2B** only one vinyl moiety inter­acts with O1*B*. The second vinyl group shows a C—H⋯O contact to O2*B* (Table 5).

## 3. Supra­molecular features

**Figure 5:**
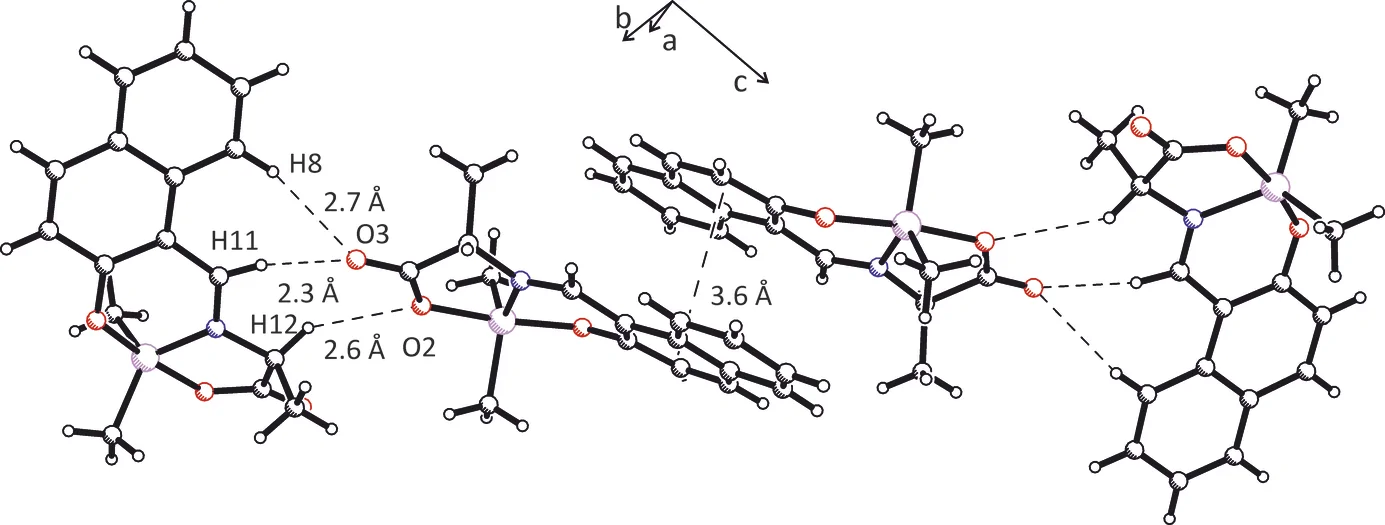
Excerpt of the mol­ecular packing in **1**: the most relevant C—H⋯O inter­actions leading to a mol­ecular zigzag chain along the *b-*axis direction are shown as well as the stacking inter­actions.

**Figure 6:**
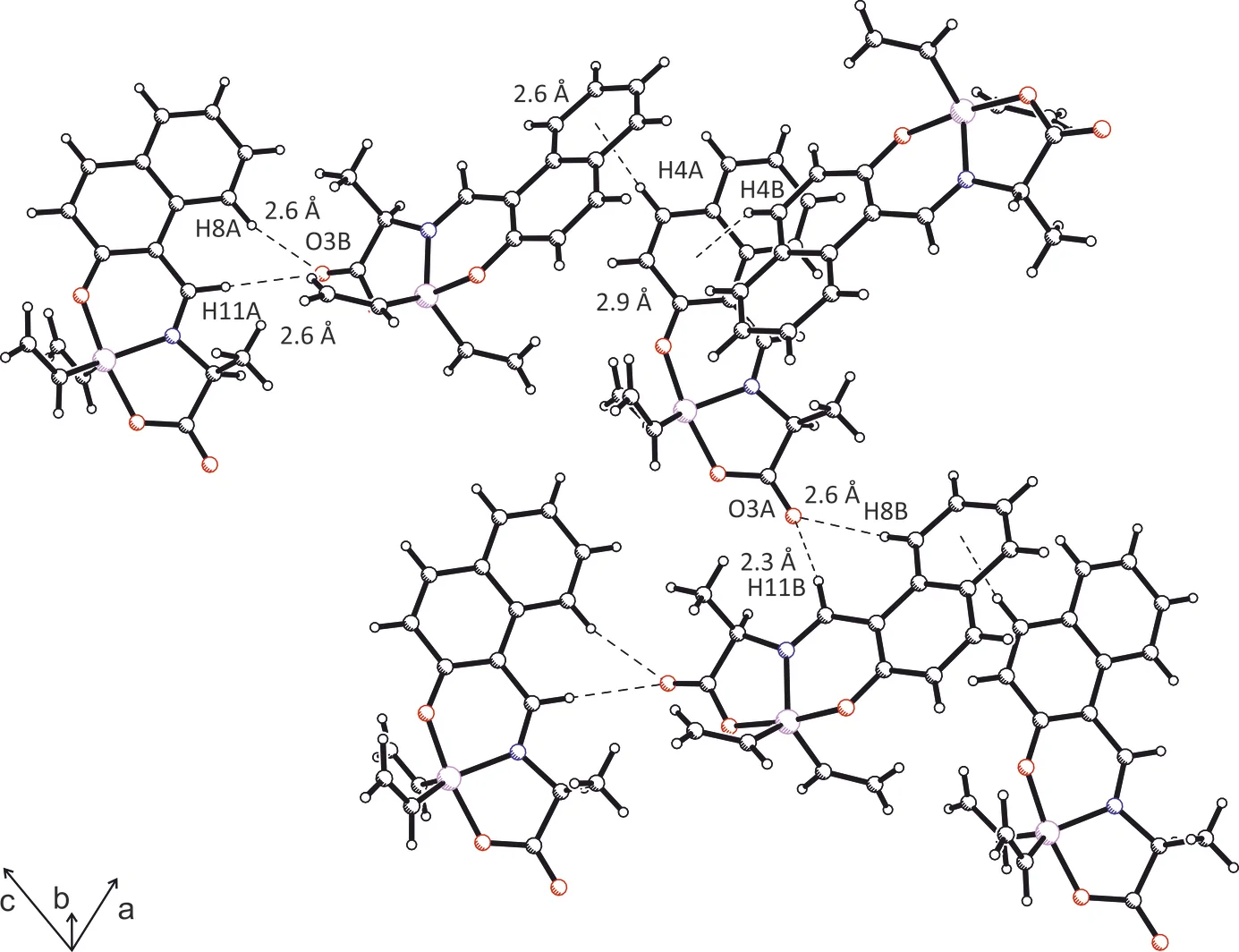
Excerpt of the mol­ecular packing in **2**: the most relevant C—H⋯O inter­actions leading to a mol­ecular zigzag chain along the *a-*axis direction are shown as well as C—H⋯π inter­actions.

**Figure 7:**
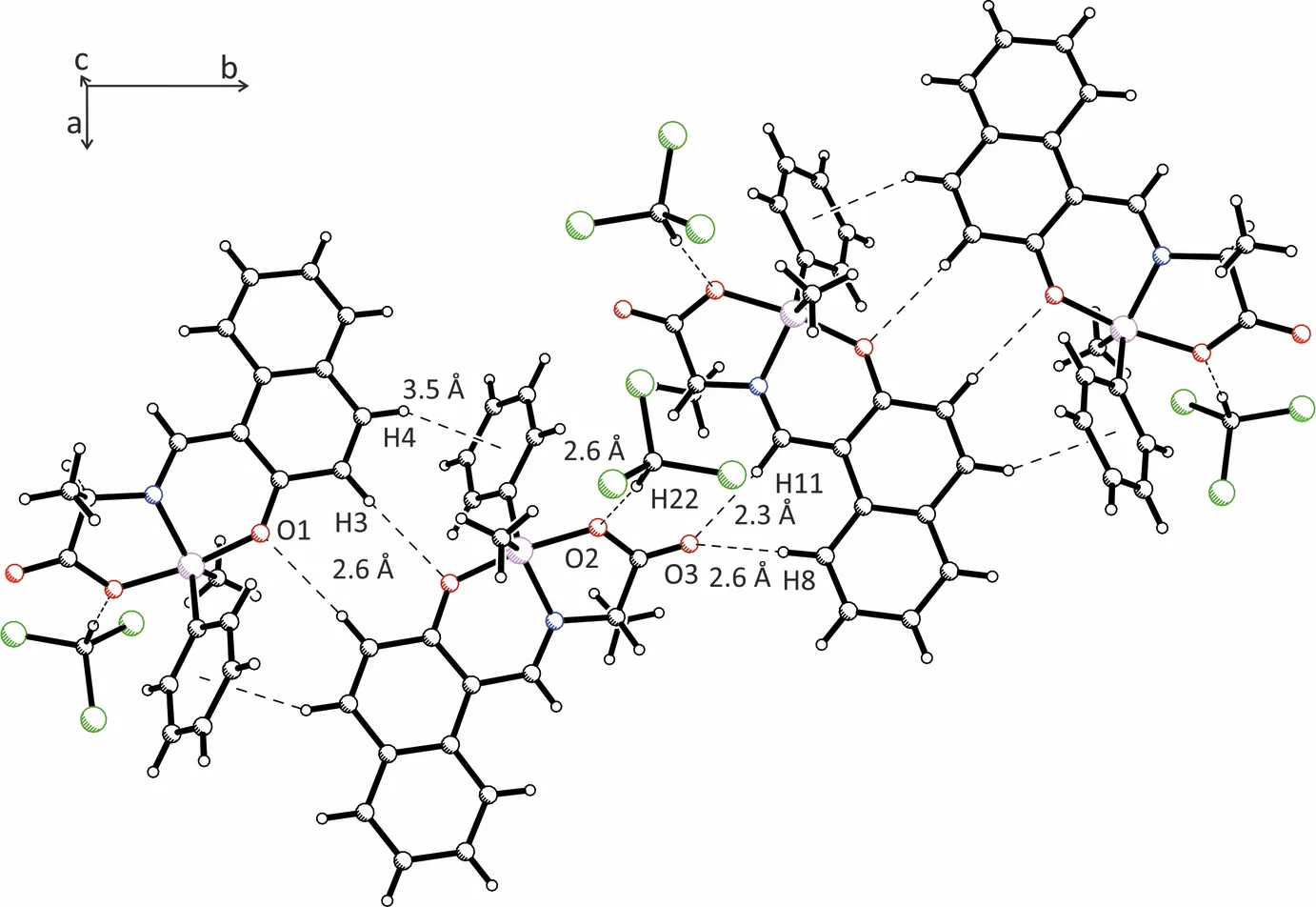
Excerpt of the mol­ecular packing in **3** along the crystallographic *c* axis. the most relevant C—H⋯O inter­actions leading to a mol­ecular zigzag chain along the *a-*axis direction are shown as well as C—H⋯π inter­actions.

The most prominent inter­molecular inter­actions in all three compounds are hydrogen bonds between the imine hydrogen atoms at C11 and the oxygen atoms O3 from neighbouring mol­ecules. The O3 oxygen atoms form bifurcated contacts, since the C8—H8 groups inter­act in C—H⋯O contacts as well. In all three structures, this combination of C—H⋯O inter­action leads to mol­ecular zigzag chains along the *b-*axis direction in **1** (Fig. 5 ▸) and along the *a-*axis directions in **2** and **3**. Only in **1** this chain is completed by the C12—H12⋯O2 contact of 2.69 Å (Table 4 ▸). Furthermore, in **1** aryl units form a mol­ecular stack between adjacent mol­ecules in a π–π inter­action with a distance of 3.5876 (8) Å while C—H⋯π inter­actions are absent. In the packing of **2**, the mentioned bifurcated C—H⋯O inter­action at O3 is combined with two C—H⋯π inter­actions of 2.63 and 2.90 Å (Table 5 ▸, Fig. 6 ▸) connecting adjacent zigzag chains, π–π stacking inter­actions are absent. In **3**, the aryl units inter­act *vice versa* compared to **1**: while π–π stacking is absent, C—H⋯π inter­actions of 2.6 Å consolidate the zigzag chains formed by the C11—H11⋯O3 contact (Table 6 ▸, Fig. 7 ▸). In contrast to **1** and **2**, the structure of **3** contains a solvent mol­ecule. These chloro­form mol­ecules are arranged in channels parallel to the crystallographic *a* axis.

In summary, all three compounds show strong and bifurcated C—H⋯O inter­actions of the imine hydrogen atom to O3, differing only in the additional contacts in the crystal packing.

## 4. Database survey

To date, 18 structurally characterized Schiff base complexes of silicon, germanium and tin have been reported in which the Schiff base ligands are derived from 2-hy­droxy­naphthaldehyde and amino acids (CSD reference codes are given with the citations below). Amino acids used for the construction of such complexes include leucine (Böhme & Fels, 2023 ▸; KORTEU), tryptophan (Lara-Cerón *et al.*, 2020 ▸; KUDPIL), valine (Schwarzer *et al.*, 2018 ▸; Böhme & Fels, 2013*a* ▸; LEQXOW, LEQXUC, ZIGTAN, ZIGTER, ZIGTIV, ZIGTOB, ZIGTUH, ZIGVAP, ZIGVET, ZIGVIX, ZIGVUJ, ZIGWAQ, ZIGWEU, ZIGWIY), glutamine (Sharma *et al.*, 2022 ▸; QAVNIO) and phenyl­glycine (Böhme & Fels, 2024 ▸; ZORWEM).

In addition, four structurally characterized silicon and tin complexes containing the *N*-salicylidenealaninato ligand have been described in the literature (Beltrán *et al.*, 2003 ▸; Böhme & Fels, 2024 ▸; Singh *et al.*, 2018 ▸; EHIZOK, EHIZUQ, FOLXEN, NEYDED). In this ligand, the naphthyl group is replaced by a phenyl group, rendering it closely related to *N*-[(2-oxidonaphthalen-1-yl)methyl­idene]alaninate. For these related penta­coordinated silicon complexes, very similar coordination geometries with distorted trigonal–bipyramidal coordination have been observed.

## 5. Synthesis and crystallization

**Ligand H_2_*****L*****:** The ligand was prepared according to a modified literature procedure (Nitta *et al.*, 1992 ▸). dl-Alanine (2.67 g, 30 mmol) was suspended in absolute EtOH (200 mL) and MeOH (50 mL). To this suspension, 2-hy­droxy-1-naphthaldehyde (6.89 g, 40 mmol) was added. The yellow suspension was stirred under reflux for 8 h. After cooling the yellow–orange reaction mixture to room temperature, the resulting yellow precipitate was collected by filtration, washed with diethyl ether (2 × 50 mL), and dried under vacuum. Yield: 6.22 g (85%), yellow solid, m.p.: 463-466 K.

^1^H NMR (DMSO-*d*_6_): δ = 1.57 (*d*, 3H, CH_3_, ^3^*J*_HH_ = 7.2 Hz), 4.57 (*q*, 1H, CH–COO, ^3^*J*_HH_ = 7.2 Hz), 6.78–8.10- (*m*, 6H, aromatic H), 9.18 (*s*, 1H, CH=N), 13.41, 14.30 (*br*, 2H, OH). ^13^C NMR (DMSO-*d*_6_): δ = 19.6 (CH_3_), 58.9 (CH—COO), 106.4 (C_Ar_—CH=N), 118.9, 122.6, 124.8, 125.7, 128.1, 129.0, 134.2, 137.3 (8 × C_Ar_), 159.0 (CH=N), 173.0 (C_Ar_—OH), 175.8 (COO).

**General Procedure for complex preparation:** The ligand H_2_*L* was placed in a reaction flask equipped with a dropping funnel. THF was added via syringe, followed by tri­ethyl­amine. The resulting solution or suspension was cooled to 273 K. The di­chloro­diorganosilane was dissolved in THF and added dropwise via the dropping funnel, during which a white precipitate formed. The reaction mixture was stirred for approximately 15 min at 273 K and then for two days at room temperature. The precipitated tri­ethyl­ammonium chloride was removed by filtration and the filter cake was washed with THF (2–3 × 10 mL). The filtrate was concentrated to dryness under reduced pressure and the residue was dissolved in CDCl_3_ (3 mL). After the NMR spectra had been recorded, *n*-hexane was added, resulting in the formation of crystals. The solid was collected by filtration, washed with *n*-hexane and dried under reduced pressure. The qu­anti­ties of reagents used for the individual compounds are given below.

Compound **1**: H_2_L (1.00 g, 4.11 mmol) was reacted with SiCl_2_Me_2_ (0.56 g, 4.32 mmol) and NEt_3_ (1.25 g, 50% excess, 12.33 mmol). Yield: 0.51 g (41%), yellow crystals, m.p.: 428–433 K (decomposition, crystals turn red); 449-451 K (remaining crystals fully melted).

^1^H NMR (CDCl_3_) δ 0.29 (*s*, 3H, Si—CH_3_), 0.57 (*s*, 3H, Si—CH_3_), 1.70 (*d*, 3H, CH–*-*CH_3_, ^3^*J*_HH_ = 7.3 Hz), 4.37 (*m*, 1H, CH–CH_3_), 6.97–8.01 (*m*, 6H, aromatic H), 9.12 (*s*, 1H, CH=N). ^13^C NMR (CDCl_3_) δ 2.4 (Si—CH_3_), 5.1 (Si—CH_3_), 21.1 (CH—CH_3_), 63.2 (CH—COO), 108.9 (C_Ar_—CH=N), 118.7, 122.2, 124.8, 127.3, 129.6, 129.7, 132.2, 141.9 (8 x C_Ar_), 163.8 (CH=N), 168.6 (C_Ar_—O), 171.8 (COO). ^29^Si NMR (CDCl_3_) δ −66.1. ^29^Si CP/MAS NMR δ −67.4 (ν_rot_ = 5 and 2 kHz).

Compound **2**: H_2_*L* (1.03 g, 4.23 mmol) was reacted with SiCl_2_Vin_2_ (0.72 g, 90% solution, 10% excess, 4.65 mmol) and NEt_3_ (1.11 g, 30% excess, 11.00 mmol). Yield: 0.91 g (66%), yellow–orange crystals, m.p.: 443–445 K.

^1^H NMR (CDCl_3_) δ 1.69 (*d*, 3H, CH—CH_3_, ^3^*J*_HH_ = 7.3 Hz), 4.39 (*dq*, 1H, CH—COO, ^3^*J*_HH_ = 7.3 Hz, ^2^*J*_HN_ = 1.0 Hz), 5.96–6.19 (*m*, 6H, vinyl H), 7.13–8.07 (*m*, 6H, aromatic H), 9.13 (*s*, 1H, CH=N). ^13^C NMR (CDCl_3_) δ 20.9 (CH—CH_3_), 63.0 (CH—COO), 109.0 (C_Ar_—CH=N), 118.7, 122.0, 125.1, 127.5, 129.8, 129.9, 132.2, 137.6, 137.7, 137.8, 139.1, 142.4 (8 × C_Ar_, 2 × Si—CH=CH_2_), 163.8 (CH=N), 168.7 (C_Ar_—O), 172.0 (COO). ^29^Si NMR (CDCl_3_) δ −101.9. ^29^Si CP/MAS δ −102.0, −104.4 (ν_rot_ = 5 and 1.5 kHz).

Compound **3**: H_2_*L* (1.51 g, 6.21 mmol) was reacted with SiCl_2_MePh (1.25 g, 50% excess, 6.52 mmol) and NEt_3_ (1.88 g, 50% excess, 18.63 mmol). Two silicon-complex mol­ecules incorporate one CHCl_3_ mol­ecule in the crystal, giving the mol­ecular formula C_43_H_39_N_2_O_6_Si_2_Cl_3_. Yield: 1.29 g (49%), yellow crystals, m.p.: 389–392 K.

^1^H NMR (CDCl_3_) δ 0.55 (*s*, 3H, Si—CH_3_), 1.53 (*d*, 3H, CH—CH_3_, ^3^*J*_HH_ = 7.4 Hz), 4.40 (*m*, 1H, CH—COO), 7.11–8.06 (*m*, 11H, aromatic H), 9.18 (*d*, 1H, CH=N, ^2^*J*_HN_ = 30.0 Hz). ^13^C NMR (CDCl_3_) δ 3.6 (CH_3_), 20.7 (CH—CH_3_), 63.3 (CH—COO), 108.8 (C_Ar_—CH=N), 118.5, 122.2, 125.1, 127.6, 127.7, 129.6, 129.8, 129.9, 132.1, 136.2, 139.5, 142.4 (13 × C_Ar_), 163.7 (CH=N), 168.7 (C_Ar_—O), 171.7 (COO). ^29^Si NMR (CDCl_3_) δ −82.7. ^29^Si CP/MAS δ −83.7 (ν_rot_ = 4 and 1.75 kHz).

## 6. Refinement

Crystal data, data collection and structure refinement details are summarized in Table 7 ▸. Hydrogen atoms bonded to C were positioned geometrically and allowed to ride on their parent atoms, with C—H = 0.95 Å for naphthyl, phenyl, and vinyl-H, 1.0 for C—H, and 0.98 Å for CH_3_. *U*_iso_(H) = *xU*_eq_(C), where *x* = 1.2 for naphthyl, phenyl, vinyl and C—H, and 1.5 for CH_3_. Hydrogen atoms at imine carbon atom C11 were localized from residual electron-density maps and were freely refined. The crystal structure of **3** contains a solvent-accessible void of 156 Å^3^. Investigation with the SQUEEZE procedure (Spek, 2015 ▸), showed that there is no electron density in the void. Refinement of a squeezed data set gave no improvements or changes in the results. Therefore, the SQUEEZE procedure was not applied, but instead the original data were used for the final refinement.

## Supplementary Material

Crystal structure: contains datablock(s) 1, 2, 3, global. DOI: 10.1107/S2056989026008406/tx2115sup1.cif

Structure factors: contains datablock(s) 1. DOI: 10.1107/S2056989026008406/tx21151sup2.hkl

Structure factors: contains datablock(s) 2. DOI: 10.1107/S2056989026008406/tx21152sup3.hkl

Structure factors: contains datablock(s) 3. DOI: 10.1107/S2056989026008406/tx21153sup4.hkl

CCDC references: 2581009, 2581010, 2581011

Additional supporting information:  crystallographic information; 3D view; checkCIF report

## Figures and Tables

**Table 1:** Selected geometric parameters (Å, °) for **1**[Chem scheme1]

Si1—O1	1.7892 (11)	Si1—O2	1.8589 (12)
Si1—N1	1.8541 (12)	Si1—C16	1.8608 (16)
Si1—C15	1.8571 (16)		
			
O1—Si1—N1	88.87 (5)	C15—Si1—O2	91.33 (7)
O1—Si1—C15	92.06 (7)	O1—Si1—C16	94.18 (7)
N1—Si1—C15	120.43 (7)	N1—Si1—C16	119.32 (7)
O1—Si1—O2	171.03 (5)	C15—Si1—C16	119.98 (8)
N1—Si1—O2	82.25 (5)	O2—Si1—C16	91.30 (7)

**Table 2:** Selected geometric parameters (Å, °) for **2**[Chem scheme1]

Si1*A*—O1*A*	1.7666 (12)	Si1*B*—O1*B*	1.7869 (12)
Si1*A*—O2*A*	1.8314 (12)	Si1*B*—O2*B*	1.8192 (12)
Si1*A*—C17*A*	1.8601 (17)	Si1*B*—N1*B*	1.8528 (14)
Si1*A*—N1*A*	1.8625 (14)	Si1*B*—C15*B*	1.8662 (17)
Si1*A*—C15*A*	1.8687 (17)	Si1*B*—C17*B*	1.8686 (19)
			
O1*A*—Si1*A*—O2*A*	169.71 (6)	O1*B*—Si1*B*—O2*B*	172.71 (6)
O1*A*—Si1*A*—C17*A*	96.47 (7)	O1*B*—Si1*B*—N1*B*	89.42 (6)
O2*A*—Si1*A*—C17*A*	92.55 (7)	O2*B*—Si1*B*—N1*B*	83.49 (6)
O1*A*—Si1*A*—N1*A*	89.15 (6)	O1*B*—Si1*B*—C15*B*	89.31 (7)
O2*A*—Si1*A*—N1*A*	82.74 (6)	O2*B*—Si1*B*—C15*B*	92.61 (7)
C17*A*—Si1*A*—N1*A*	113.14 (7)	N1*B*—Si1*B*—C15*B*	118.46 (7)
O1*A*—Si1*A*—C15*A*	91.69 (7)	O1*B*—Si1*B*—C17*B*	94.97 (8)
O2*A*—Si1*A*—C15*A*	88.27 (7)	O2*B*—Si1*B*—C17*B*	89.93 (8)
C17*A*—Si1*A*—C15*A*	118.83 (7)	N1*B*—Si1*B*—C17*B*	118.97 (7)
N1*A*—Si1*A*—C15*A*	127.55 (7)	C15*B*—Si1*B*—C17*B*	122.43 (8)

**Table 3:** Selected geometric parameters (Å, °) for **3**[Chem scheme1]

Si1—O1	1.7879 (13)	Si1—C15	1.863 (2)
Si1—O2	1.8522 (14)	Si1—C16	1.8857 (16)
Si1—N1	1.8534 (14)		
			
O1—Si1—O2	169.99 (6)	N1—Si1—C15	117.61 (8)
O1—Si1—N1	88.28 (6)	O1—Si1—C16	91.95 (6)
O2—Si1—N1	82.06 (6)	O2—Si1—C16	90.90 (7)
O1—Si1—C15	93.89 (10)	N1—Si1—C16	121.59 (7)
O2—Si1—C15	92.92 (10)	C15—Si1—C16	120.63 (8)

**Table 4:** Hydrogen-bond geometry (Å, °) for **1**[Chem scheme1]

*D*—H⋯*A*	*D*—H	H⋯*A*	*D*⋯*A*	*D*—H⋯*A*
C8—H8⋯O3^i^	0.95	2.58	3.440 (2)	150
C11—H11⋯O3^i^	0.956 (16)	2.266 (16)	3.1882 (18)	162.0 (13)
C12—H12⋯O2^i^	1.00	2.69	3.4882 (17)	137

Symmetry code: (i) 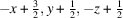.

**Table 5:** Hydrogen-bond geometry (Å, °) for **2**[Chem scheme1] *Cg*3 and *Cg*14 are the centroids of the C1*A*–C4*A*,C9*A*,C10*A* and C5*B*–C10*B* rings, respectively.

*D*—H⋯*A*	*D*—H	H⋯*A*	*D*⋯*A*	*D*—H⋯*A*
C8*A*—H8*A*⋯O3*B*^i^	0.95	2.56	3.429 (2)	152
C11*A*—H11*A*⋯O3*B*^i^	0.97 (2)	2.56 (2)	3.526 (2)	172.3 (17)
C12*A*—H12*A*⋯O3*B*^ii^	1.04 (2)	2.53 (2)	3.370 (2)	138.0 (18)
C16*A*—H16*A*⋯O1*A*	0.95	2.39	2.829 (2)	108
C18*A*—H18*A*⋯O1*A*	0.95	2.45	2.911 (2)	110
C8*B*—H8*B*⋯O3*A*^iii^	0.95	2.56	3.479 (2)	163
C11*B*—H11*B*⋯O3*A*^iii^	0.979 (19)	2.30 (2)	3.260 (2)	165.7 (15)
C16*B*—H16*C*⋯O2*B*	0.95	2.58	2.937 (2)	103
C18*B*—H18*C*⋯O1*B*	0.95	2.46	2.917 (2)	109
C4*A*—H4*A*⋯*Cg*14	0.95	2.63	3.3641 (18)	134
C4*B*—H4*B*⋯*Cg*3^iv^	0.95	2.90	3.744 (2)	149

Symmetry codes: (i) 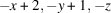; (ii) 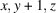; (iii) 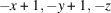; (iv) 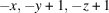.

**Table 6:** Hydrogen-bond geometry (Å, °) for **3**[Chem scheme1] *Cg*5 is the centroid of the C16–C21 ring.

*D*—H⋯*A*	*D*—H	H⋯*A*	*D*⋯*A*	*D*—H⋯*A*
C3—H3⋯O1^i^	0.95	2.62	3.562 (2)	171
C8—H8⋯O3^ii^	0.95	2.64	3.530 (3)	155
C11—H11⋯O3^ii^	0.95	2.34	3.278 (2)	169
C22—H22⋯O2^iii^	1.00	2.55	3.534 (5)	169
C4—H4⋯*Cg*5^i^	0.95	2.67	3.506 (2)	147

Symmetry codes: (i) 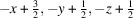; (ii) 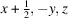; (iii) 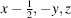.

**Table 7:** Experimental details

	**1**	**2**	**3**
Crystal data			
Chemical formula	C_16_H_17_NO_3_Si	C_18_H_17_NO_3_Si	2C_21_H_19_NO_3_Si·CHCl_3_
*M*_r_	299.39	323.41	842.29
Crystal system, space group	Monoclinic, *P*2_1_/*n*	Triclinic, *P* 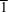	Monoclinic, *I*2/*a*
Temperature (K)	153	153	153
*a*, *b*, *c* (Å)	8.0857 (2), 10.3710 (3), 18.0590 (6)	11.1966 (5), 11.8862 (5), 14.1113 (7)	10.7122 (5), 27.2203 (13), 14.1716 (8)
α, β, γ (°)	90, 101.641 (2), 90	68.634 (3), 72.438 (4), 66.638 (3)	90, 93.546 (4), 90
*V* (Å^3^)	1483.22 (8)	1578.15 (14)	4124.4 (4)
*Z*	4	4	4
Radiation type	Mo *K*α	Mo *K*α	Mo *K*α
μ (mm^−1^)	0.17	0.16	0.33
Crystal size (mm)	0.30 × 0.28 × 0.15	0.18 × 0.17 × 0.15	0.40 × 0.35 × 0.30

Data collection			
Diffractometer	Bruker SMART CCD area-detector	Stoe IPDS 2	Stoe *IPDS* 2T
Absorption correction	Multi-scan (*SADABS*; Krause *et al.*, 2015 ▸)	Integration (*X-RED*; Stoe, 2024 ▸)	Integration (*X-RED*; Stoe, 2024 ▸)
*T*_min_, *T*_max_	0.951, 0.975	0.951, 0.987	0.733, 0.773
No. of measured, independent and observed [*I* > 2σ(*I*)] reflections	21377, 3406, 2821	21449, 7237, 6364	25032, 4732, 3956
*R*_int_	0.030	0.036	0.027

Refinement			
*R*[*F*^2^ > 2σ(*F*^2^)], *wR*(*F*^2^), *S*	0.036, 0.103, 1.02	0.041, 0.102, 1.10	0.044, 0.118, 1.06
No. of reflections	3406	7237	4732
No. of parameters	197	433	273
No. of restraints	0	0	27
H-atom treatment	H atoms treated by a mixture of independent and constrained refinement	H atoms treated by a mixture of independent and constrained refinement	H-atom parameters constrained
Δρ_max_, Δρ_min_ (e Å^−3^)	0.31, −0.29	0.45, −0.36	0.53, −0.47

Computer programs: *SMART* and *SAINT* (Bruker, 2004 ▸), *X-AREA* and *X-RED* (Stoe, 2024 ▸), *SHELXT* (Sheldrick, 2015*a* ▸), *SHELXL2019/2* (Sheldrick, 2015*b* ▸) and *ORTEP-3 for Windows* (Farrugia, 2012 ▸).

## supplementary crystallographic information

### (RS)-Dimethyl{N-[(2-oxidonaphthalen-1-yl)methylidene]\ alaninato}silicon (1) Crystal data

**Table:**

C_16_H_17_NO_3_Si	*D*_x_ = 1.341 Mg m^−^^3^
*M**_r_* = 299.39	Melting point: 433 K
Monoclinic, *P*2_1_/*n*	Mo *K*α radiation, λ = 0.71073 Å
*a* = 8.0857 (2) Å	Cell parameters from 9936 reflections
*b* = 10.3710 (3) Å	θ = 2.3–32.4°
*c* = 18.0590 (6) Å	µ = 0.17 mm^−^^1^
β = 101.641 (2)°	*T* = 153 K
*V* = 1483.22 (8) Å^3^	Prism, yellow
*Z* = 4	0.30 × 0.28 × 0.15 mm
*F*(000) = 632	

### (RS)-Dimethyl{N-[(2-oxidonaphthalen-1-yl)methylidene]\ alaninato}silicon (1) Data collection

**Table:**

Bruker SMART CCD area-detector diffractometer	3406 independent reflections
Radiation source: sealed tube	2821 reflections with *I* > 2σ(*I*)
Graphite monochromator	*R*_int_ = 0.030
phi and ω scans	θ_max_ = 27.5°, θ_min_ = 2.3°
Absorption correction: multi-scan (SADABS; Krause *et al.*, 2015)	*h* = −10→10
*T*_min_ = 0.951, *T*_max_ = 0.975	*k* = −13→13
21377 measured reflections	*l* = −23→23

### (RS)-Dimethyl{N-[(2-oxidonaphthalen-1-yl)methylidene]\ alaninato}silicon (1) Refinement

**Table:**

Refinement on *F*^2^	Primary atom site location: structure-invariant direct methods
Least-squares matrix: full	Secondary atom site location: difference Fourier map
*R*[*F*^2^ > 2σ(*F*^2^)] = 0.036	Hydrogen site location: mixed
*wR*(*F*^2^) = 0.103	H atoms treated by a mixture of independent and constrained refinement
*S* = 1.02	*w* = 1/[σ^2^(*F*_o_^2^) + (0.0555*P*)^2^ + 0.4849*P*] where *P* = (*F*_o_^2^ + 2*F*_c_^2^)/3
3406 reflections	(Δ/σ)_max_ < 0.001
197 parameters	Δρ_max_ = 0.31 e Å^−^^3^
0 restraints	Δρ_min_ = −0.28 e Å^−^^3^

### (RS)-Dimethyl{N-[(2-oxidonaphthalen-1-yl)methylidene]\ alaninato}silicon (1) Special details

**Table:**

Geometry. All esds (except the esd in the dihedral angle between two l.s. planes) are estimated using the full covariance matrix. The cell esds are taken into account individually in the estimation of esds in distances, angles and torsion angles; correlations between esds in cell parameters are only used when they are defined by crystal symmetry. An approximate (isotropic) treatment of cell esds is used for estimating esds involving l.s. planes.

### (RS)-Dimethyl{N-[(2-oxidonaphthalen-1-yl)methylidene]\ alaninato}silicon (1) Fractional atomic coordinates and isotropic or equivalent isotropic displacement parameters (Å^2^)

**Table:**

	*x*	*y*	*z*	*U*_iso_*/*U*_eq_	
Si1	0.89958 (5)	0.11805 (4)	0.11488 (2)	0.02429 (12)	
O1	0.97215 (13)	0.19047 (9)	0.03733 (6)	0.0266 (2)	
O2	0.82199 (15)	0.06904 (10)	0.20085 (6)	0.0347 (3)	
O3	0.73054 (18)	0.13472 (11)	0.30340 (7)	0.0462 (3)	
N1	0.84982 (14)	0.28130 (11)	0.14610 (7)	0.0217 (2)	
C1	0.81870 (16)	0.38675 (13)	0.02463 (8)	0.0210 (3)	
C2	0.90754 (17)	0.28927 (13)	−0.00437 (8)	0.0228 (3)	
C3	0.93528 (19)	0.29921 (14)	−0.07950 (8)	0.0276 (3)	
H3	1.002252	0.236597	−0.098181	0.033*	
C4	0.86608 (19)	0.39810 (15)	−0.12445 (8)	0.0299 (3)	
H4	0.882329	0.401402	−0.175064	0.036*	
C5	0.6989 (2)	0.59953 (16)	−0.14610 (9)	0.0346 (4)	
H5	0.710956	0.600365	−0.197344	0.042*	
C6	0.61357 (19)	0.69689 (16)	−0.11982 (10)	0.0364 (4)	
H6	0.565886	0.764760	−0.152603	0.044*	
C7	0.59646 (19)	0.69643 (14)	−0.04406 (10)	0.0318 (3)	
H7	0.538933	0.765319	−0.025463	0.038*	
C8	0.66184 (18)	0.59762 (14)	0.00357 (9)	0.0270 (3)	
H8	0.647932	0.598563	0.054560	0.032*	
C9	0.74935 (16)	0.49477 (13)	−0.02223 (8)	0.0220 (3)	
C10	0.76989 (17)	0.49718 (14)	−0.09837 (8)	0.0265 (3)	
C11	0.80745 (16)	0.38095 (13)	0.10179 (8)	0.0215 (3)	
H11	0.7699 (19)	0.4536 (16)	0.1265 (9)	0.022 (4)*	
C12	0.82703 (18)	0.29168 (13)	0.22505 (8)	0.0244 (3)	
H12	0.729191	0.349551	0.227204	0.029*	
C13	0.7871 (2)	0.15601 (15)	0.24710 (9)	0.0314 (3)	
C14	0.9845 (2)	0.34254 (16)	0.27807 (9)	0.0338 (4)	
H14A	1.081565	0.288518	0.273790	0.051*	
H14B	0.967324	0.340281	0.330249	0.051*	
H14C	1.005806	0.431496	0.264269	0.051*	
C15	1.1090 (2)	0.04395 (15)	0.15188 (10)	0.0351 (4)	
H15A	1.167624	0.029570	0.110090	0.053*	
H15B	1.093669	−0.038636	0.175982	0.053*	
H15C	1.176357	0.101820	0.189089	0.053*	
C16	0.7309 (2)	0.02255 (17)	0.05338 (10)	0.0387 (4)	
H16A	0.677398	0.075077	0.010026	0.058*	
H16B	0.646072	−0.003066	0.082365	0.058*	
H16C	0.780538	−0.054652	0.035368	0.058*	

### (RS)-Dimethyl{N-[(2-oxidonaphthalen-1-yl)methylidene]\ alaninato}silicon (1) Atomic displacement parameters (Å^2^)

**Table:**

	*U*^11^	*U*^22^	*U*^33^	*U*^12^	*U*^13^	*U*^23^
Si1	0.0289 (2)	0.0183 (2)	0.0255 (2)	0.00074 (15)	0.00502 (15)	−0.00159 (15)
O1	0.0306 (5)	0.0244 (5)	0.0258 (5)	0.0057 (4)	0.0079 (4)	−0.0007 (4)
O2	0.0531 (7)	0.0204 (5)	0.0335 (6)	−0.0039 (5)	0.0158 (5)	0.0012 (4)
O3	0.0765 (9)	0.0329 (6)	0.0368 (7)	−0.0060 (6)	0.0294 (7)	0.0065 (5)
N1	0.0253 (6)	0.0199 (5)	0.0209 (6)	−0.0007 (4)	0.0068 (4)	−0.0012 (4)
C1	0.0202 (6)	0.0207 (6)	0.0223 (7)	−0.0033 (5)	0.0046 (5)	−0.0015 (5)
C2	0.0216 (6)	0.0233 (7)	0.0227 (7)	−0.0029 (5)	0.0030 (5)	−0.0025 (5)
C3	0.0305 (7)	0.0286 (8)	0.0251 (8)	−0.0034 (6)	0.0087 (6)	−0.0061 (6)
C4	0.0335 (8)	0.0372 (8)	0.0193 (7)	−0.0077 (6)	0.0059 (6)	−0.0022 (6)
C5	0.0352 (8)	0.0405 (9)	0.0255 (8)	−0.0062 (7)	−0.0004 (6)	0.0083 (7)
C6	0.0306 (8)	0.0325 (9)	0.0412 (10)	−0.0034 (6)	−0.0042 (7)	0.0138 (7)
C7	0.0254 (7)	0.0252 (8)	0.0428 (9)	−0.0011 (6)	0.0020 (6)	0.0042 (6)
C8	0.0253 (7)	0.0251 (7)	0.0303 (8)	−0.0013 (5)	0.0047 (6)	0.0019 (6)
C9	0.0185 (6)	0.0226 (7)	0.0239 (7)	−0.0049 (5)	0.0020 (5)	0.0009 (5)
C10	0.0245 (7)	0.0296 (7)	0.0240 (8)	−0.0073 (6)	0.0012 (5)	0.0014 (6)
C11	0.0217 (6)	0.0187 (6)	0.0248 (7)	−0.0017 (5)	0.0061 (5)	−0.0022 (5)
C12	0.0320 (7)	0.0219 (7)	0.0213 (7)	−0.0004 (5)	0.0101 (6)	0.0012 (5)
C13	0.0404 (9)	0.0263 (8)	0.0283 (8)	−0.0029 (6)	0.0091 (7)	0.0033 (6)
C14	0.0411 (9)	0.0353 (8)	0.0243 (8)	−0.0052 (7)	0.0054 (6)	−0.0036 (6)
C15	0.0388 (8)	0.0285 (8)	0.0361 (9)	0.0072 (7)	0.0031 (7)	0.0026 (7)
C16	0.0380 (9)	0.0338 (9)	0.0432 (10)	−0.0060 (7)	0.0055 (7)	−0.0098 (7)

### (RS)-Dimethyl{N-[(2-oxidonaphthalen-1-yl)methylidene]\ alaninato}silicon (1) Geometric parameters (Å, º)

**Table:**

Si1—O1	1.7892 (11)	C6—C7	1.403 (2)
Si1—N1	1.8541 (12)	C6—H6	0.9500
Si1—C15	1.8571 (16)	C7—C8	1.373 (2)
Si1—O2	1.8589 (12)	C7—H7	0.9500
Si1—C16	1.8608 (16)	C8—C9	1.410 (2)
O1—C2	1.3157 (17)	C8—H8	0.9500
O2—C13	1.298 (2)	C9—C10	1.418 (2)
O3—C13	1.2161 (19)	C11—H11	0.956 (16)
N1—C11	1.3096 (18)	C12—C13	1.515 (2)
N1—C12	1.4780 (17)	C12—C14	1.524 (2)
C1—C2	1.4015 (19)	C12—H12	1.0000
C1—C11	1.4158 (19)	C14—H14A	0.9800
C1—C9	1.4471 (19)	C14—H14B	0.9800
C2—C3	1.423 (2)	C14—H14C	0.9800
C3—C4	1.357 (2)	C15—H15A	0.9800
C3—H3	0.9500	C15—H15B	0.9800
C4—C10	1.425 (2)	C15—H15C	0.9800
C4—H4	0.9500	C16—H16A	0.9800
C5—C6	1.361 (2)	C16—H16B	0.9800
C5—C10	1.414 (2)	C16—H16C	0.9800
C5—H5	0.9500		
			
O1—Si1—N1	88.87 (5)	C9—C8—H8	119.6
O1—Si1—C15	92.06 (7)	C8—C9—C10	118.15 (13)
N1—Si1—C15	120.43 (7)	C8—C9—C1	123.60 (13)
O1—Si1—O2	171.03 (5)	C10—C9—C1	118.25 (13)
N1—Si1—O2	82.25 (5)	C5—C10—C9	119.45 (14)
C15—Si1—O2	91.33 (7)	C5—C10—C4	121.43 (14)
O1—Si1—C16	94.18 (7)	C9—C10—C4	119.09 (13)
N1—Si1—C16	119.32 (7)	N1—C11—C1	125.03 (13)
C15—Si1—C16	119.98 (8)	N1—C11—H11	113.9 (9)
O2—Si1—C16	91.30 (7)	C1—C11—H11	121.0 (9)
C2—O1—Si1	128.30 (9)	N1—C12—C13	105.11 (11)
C13—O2—Si1	120.08 (10)	N1—C12—C14	112.35 (12)
C11—N1—C12	117.63 (12)	C13—C12—C14	110.58 (13)
C11—N1—Si1	125.51 (10)	N1—C12—H12	109.6
C12—N1—Si1	115.90 (9)	C13—C12—H12	109.6
C2—C1—C11	118.47 (13)	C14—C12—H12	109.6
C2—C1—C9	120.47 (13)	O3—C13—O2	125.37 (15)
C11—C1—C9	120.94 (12)	O3—C13—C12	121.64 (14)
O1—C2—C1	121.48 (13)	O2—C13—C12	112.99 (13)
O1—C2—C3	118.78 (12)	C12—C14—H14A	109.5
C1—C2—C3	119.70 (13)	C12—C14—H14B	109.5
C4—C3—C2	120.00 (14)	H14A—C14—H14B	109.5
C4—C3—H3	120.0	C12—C14—H14C	109.5
C2—C3—H3	120.0	H14A—C14—H14C	109.5
C3—C4—C10	122.29 (14)	H14B—C14—H14C	109.5
C3—C4—H4	118.9	Si1—C15—H15A	109.5
C10—C4—H4	118.9	Si1—C15—H15B	109.5
C6—C5—C10	121.09 (15)	H15A—C15—H15B	109.5
C6—C5—H5	119.5	Si1—C15—H15C	109.5
C10—C5—H5	119.5	H15A—C15—H15C	109.5
C5—C6—C7	119.62 (15)	H15B—C15—H15C	109.5
C5—C6—H6	120.2	Si1—C16—H16A	109.5
C7—C6—H6	120.2	Si1—C16—H16B	109.5
C8—C7—C6	120.81 (15)	H16A—C16—H16B	109.5
C8—C7—H7	119.6	Si1—C16—H16C	109.5
C6—C7—H7	119.6	H16A—C16—H16C	109.5
C7—C8—C9	120.86 (15)	H16B—C16—H16C	109.5
C7—C8—H8	119.6		
			
N1—Si1—O1—C2	−38.93 (12)	C7—C8—C9—C1	−178.91 (13)
C15—Si1—O1—C2	−159.35 (12)	C2—C1—C9—C8	−178.58 (13)
C16—Si1—O1—C2	80.39 (12)	C11—C1—C9—C8	−2.4 (2)
N1—Si1—O2—C13	−7.04 (12)	C2—C1—C9—C10	1.67 (19)
C15—Si1—O2—C13	113.51 (13)	C11—C1—C9—C10	177.81 (12)
C16—Si1—O2—C13	−126.46 (13)	C6—C5—C10—C9	1.1 (2)
O1—Si1—N1—C11	29.07 (12)	C6—C5—C10—C4	−177.04 (14)
C15—Si1—N1—C11	120.80 (12)	C8—C9—C10—C5	−1.7 (2)
O2—Si1—N1—C11	−152.22 (12)	C1—C9—C10—C5	178.06 (12)
C16—Si1—N1—C11	−65.09 (14)	C8—C9—C10—C4	176.47 (12)
O1—Si1—N1—C12	−162.45 (10)	C1—C9—C10—C4	−3.76 (19)
C15—Si1—N1—C12	−70.72 (12)	C3—C4—C10—C5	179.95 (14)
O2—Si1—N1—C12	16.26 (9)	C3—C4—C10—C9	1.8 (2)
C16—Si1—N1—C12	103.39 (11)	C12—N1—C11—C1	−179.05 (12)
Si1—O1—C2—C1	28.69 (19)	Si1—N1—C11—C1	−10.74 (19)
Si1—O1—C2—C3	−153.98 (10)	C2—C1—C11—N1	−11.6 (2)
C11—C1—C2—O1	3.52 (19)	C9—C1—C11—N1	172.20 (12)
C9—C1—C2—O1	179.75 (12)	C11—N1—C12—C13	148.52 (12)
C11—C1—C2—C3	−173.80 (12)	Si1—N1—C12—C13	−20.91 (14)
C9—C1—C2—C3	2.44 (19)	C11—N1—C12—C14	−91.17 (15)
O1—C2—C3—C4	178.12 (13)	Si1—N1—C12—C14	99.39 (12)
C1—C2—C3—C4	−4.5 (2)	Si1—O2—C13—O3	177.44 (14)
C2—C3—C4—C10	2.4 (2)	Si1—O2—C13—C12	−3.58 (18)
C10—C5—C6—C7	0.4 (2)	N1—C12—C13—O3	−166.00 (15)
C5—C6—C7—C8	−1.3 (2)	C14—C12—C13—O3	72.5 (2)
C6—C7—C8—C9	0.7 (2)	N1—C12—C13—O2	14.97 (17)
C7—C8—C9—C10	0.8 (2)	C14—C12—C13—O2	−106.50 (15)

### (RS)-Dimethyl{N-[(2-oxidonaphthalen-1-yl)methylidene]\ alaninato}silicon (1) Hydrogen-bond geometry (Å, º)

**Table:**

*D*—H···*A*	*D*—H	H···*A*	*D*···*A*	*D*—H···*A*
C8—H8···O3^i^	0.95	2.58	3.440 (2)	150
C11—H11···O3^i^	0.956 (16)	2.266 (16)	3.1882 (18)	162.0 (13)
C12—H12···O2^i^	1.00	2.69	3.4882 (17)	137

Symmetry code: (i) −*x*+3/2, *y*+1/2, −*z*+1/2.

### (RS)-Diethenyl{N-[(2-oxidonaphthalen-1-yl)methylidene]\ alaninato}silicon (2) Crystal data

**Table:**

C_18_H_17_NO_3_Si	*F*(000) = 680
*M**_r_* = 323.41	*D*_x_ = 1.361 Mg m^−^^3^
Triclinic, *P*1	Melting point: 445 K
*a* = 11.1966 (5) Å	Mo *K*α radiation, λ = 0.71073 Å
*b* = 11.8862 (5) Å	Cell parameters from 21449 reflections
*c* = 14.1113 (7) Å	θ = 1.6–29.5°
α = 68.634 (3)°	µ = 0.16 mm^−^^1^
β = 72.438 (4)°	*T* = 153 K
γ = 66.638 (3)°	Prism, yellow
*V* = 1578.15 (14) Å^3^	0.18 × 0.17 × 0.15 mm
*Z* = 4	

### (RS)-Diethenyl{N-[(2-oxidonaphthalen-1-yl)methylidene]\ alaninato}silicon (2) Data collection

**Table:**

Stoe IPDS 2 diffractometer	7237 independent reflections
Radiation source: fine-focus sealed tube	6364 reflections with *I* > 2σ(*I*)
Graphite monochromator	*R*_int_ = 0.036
rotation method, ω scans	θ_max_ = 27.5°, θ_min_ = 1.6°
Absorption correction: integration (X-RED; Stoe, 2024)	*h* = −14→14
*T*_min_ = 0.951, *T*_max_ = 0.987	*k* = −15→14
21449 measured reflections	*l* = −18→17

### (RS)-Diethenyl{N-[(2-oxidonaphthalen-1-yl)methylidene]\ alaninato}silicon (2) Refinement

**Table:**

Refinement on *F*^2^	Primary atom site location: structure-invariant direct methods
Least-squares matrix: full	Secondary atom site location: difference Fourier map
*R*[*F*^2^ > 2σ(*F*^2^)] = 0.041	Hydrogen site location: mixed
*wR*(*F*^2^) = 0.102	H atoms treated by a mixture of independent and constrained refinement
*S* = 1.10	*w* = 1/[σ^2^(*F*_o_^2^) + (0.0349*P*)^2^ + 1.2928*P*] where *P* = (*F*_o_^2^ + 2*F*_c_^2^)/3
7237 reflections	(Δ/σ)_max_ = 0.001
433 parameters	Δρ_max_ = 0.45 e Å^−^^3^
0 restraints	Δρ_min_ = −0.36 e Å^−^^3^

### (RS)-Diethenyl{N-[(2-oxidonaphthalen-1-yl)methylidene]\ alaninato}silicon (2) Special details

**Table:**

Geometry. All esds (except the esd in the dihedral angle between two l.s. planes) are estimated using the full covariance matrix. The cell esds are taken into account individually in the estimation of esds in distances, angles and torsion angles; correlations between esds in cell parameters are only used when they are defined by crystal symmetry. An approximate (isotropic) treatment of cell esds is used for estimating esds involving l.s. planes.

### (RS)-Diethenyl{N-[(2-oxidonaphthalen-1-yl)methylidene]\ alaninato}silicon (2) Fractional atomic coordinates and isotropic or equivalent isotropic displacement parameters (Å^2^)

**Table:**

	*x*	*y*	*z*	*U*_iso_*/*U*_eq_	
Si1A	0.61592 (4)	0.82607 (4)	0.24755 (3)	0.01472 (10)	
O1A	0.68683 (11)	0.78427 (11)	0.35679 (8)	0.0173 (2)	
O2A	0.57167 (12)	0.85219 (12)	0.12464 (9)	0.0234 (3)	
O3A	0.64503 (13)	0.84587 (13)	−0.03974 (10)	0.0272 (3)	
N1A	0.77600 (13)	0.83988 (13)	0.16264 (10)	0.0170 (3)	
C1A	0.87323 (14)	0.85736 (14)	0.28609 (11)	0.0147 (3)	
C2A	0.78123 (15)	0.81407 (14)	0.36978 (12)	0.0149 (3)	
C3A	0.79116 (16)	0.79517 (16)	0.47248 (12)	0.0201 (3)	
H3A	0.729126	0.764919	0.528991	0.024*	
C4A	0.88981 (17)	0.82043 (17)	0.49022 (12)	0.0224 (3)	
H4A	0.895045	0.807825	0.559501	0.027*	
C5A	1.08878 (19)	0.8874 (2)	0.42848 (14)	0.0314 (4)	
H5A	1.094945	0.872119	0.498048	0.038*	
C6A	1.1801 (2)	0.9309 (2)	0.34894 (16)	0.0380 (5)	
H6A	1.249474	0.945390	0.363465	0.046*	
C7A	1.1714 (2)	0.9538 (2)	0.24636 (15)	0.0357 (5)	
H7A	1.234738	0.984502	0.191596	0.043*	
C8A	1.07222 (18)	0.93257 (19)	0.22364 (13)	0.0264 (4)	
H8A	1.067390	0.949340	0.153491	0.032*	
C9A	0.97743 (15)	0.88592 (15)	0.30406 (12)	0.0169 (3)	
C10A	0.98532 (16)	0.86520 (17)	0.40775 (13)	0.0204 (3)	
C11A	0.86935 (15)	0.86020 (15)	0.18595 (12)	0.0167 (3)	
H11A	0.941 (2)	0.8771 (19)	0.1293 (16)	0.023 (5)*	
C12A	0.79361 (18)	0.84276 (19)	0.05363 (13)	0.0247 (4)	
H12A	0.815 (2)	0.925 (2)	0.0041 (19)	0.038 (6)*	
C13A	0.66158 (17)	0.84704 (16)	0.04150 (13)	0.0203 (3)	
C14A	0.9068 (2)	0.7296 (3)	0.02816 (19)	0.0450 (6)	
H14A	0.989897	0.734379	0.033284	0.068*	
H14B	0.911353	0.730015	−0.042427	0.068*	
H14C	0.892699	0.650674	0.077061	0.068*	
C15A	0.56096 (17)	0.68207 (17)	0.30095 (14)	0.0227 (3)	
H15A	0.506744	0.676807	0.263354	0.027*	
C16A	0.58864 (18)	0.58641 (17)	0.38505 (15)	0.0273 (4)	
H16A	0.642436	0.586348	0.425829	0.033*	
H16B	0.554695	0.517892	0.404546	0.033*	
C17A	0.49443 (16)	0.98192 (16)	0.26398 (13)	0.0198 (3)	
H17A	0.440920	1.032224	0.212695	0.024*	
C18A	0.47617 (18)	1.02887 (17)	0.34153 (14)	0.0263 (4)	
H18A	0.527602	0.981672	0.394479	0.032*	
H18B	0.411712	1.109593	0.343962	0.032*	
Si1B	0.64471 (4)	0.32792 (4)	0.19710 (3)	0.01585 (10)	
O1B	0.49557 (11)	0.43653 (11)	0.24695 (9)	0.0209 (2)	
O2B	0.78445 (11)	0.21727 (11)	0.13567 (9)	0.0215 (2)	
O3B	0.85879 (12)	0.11070 (12)	0.01694 (10)	0.0262 (3)	
N1B	0.54780 (12)	0.29066 (13)	0.13360 (10)	0.0156 (3)	
C1B	0.32824 (15)	0.37536 (14)	0.22799 (12)	0.0147 (3)	
C2B	0.37085 (15)	0.44309 (15)	0.26710 (12)	0.0170 (3)	
C3B	0.27731 (17)	0.52598 (16)	0.32657 (14)	0.0228 (3)	
H3B	0.305911	0.572849	0.352230	0.027*	
C4B	0.14647 (17)	0.53791 (16)	0.34661 (14)	0.0231 (3)	
H4B	0.084856	0.595137	0.384980	0.028*	
C5B	−0.03648 (16)	0.47764 (18)	0.33682 (14)	0.0261 (4)	
H5B	−0.097792	0.535006	0.375168	0.031*	
C6B	−0.08048 (17)	0.40592 (19)	0.30637 (15)	0.0282 (4)	
H6B	−0.171733	0.413842	0.323426	0.034*	
C7B	0.00958 (16)	0.32098 (18)	0.25006 (13)	0.0242 (4)	
H7B	−0.021012	0.270889	0.229561	0.029*	
C8B	0.14236 (15)	0.30920 (16)	0.22398 (12)	0.0188 (3)	
H8B	0.202203	0.250810	0.186057	0.023*	
C9B	0.19004 (15)	0.38320 (14)	0.25319 (11)	0.0150 (3)	
C10B	0.09904 (15)	0.46729 (15)	0.31187 (13)	0.0191 (3)	
C11B	0.42027 (15)	0.30840 (14)	0.15682 (12)	0.0152 (3)	
H11B	0.3871 (18)	0.2753 (18)	0.1193 (15)	0.017 (5)*	
C12B	0.62616 (15)	0.21943 (16)	0.05570 (13)	0.0193 (3)	
H12B	0.596 (2)	0.142 (2)	0.0686 (16)	0.026 (5)*	
C13B	0.76916 (15)	0.17648 (15)	0.06785 (12)	0.0183 (3)	
C14B	0.60881 (18)	0.30060 (19)	−0.05340 (14)	0.0283 (4)	
H14D	0.516889	0.323690	−0.060230	0.042*	
H14E	0.668051	0.252359	−0.102560	0.042*	
H14F	0.630248	0.378322	−0.068097	0.042*	
C15B	0.71082 (16)	0.46356 (16)	0.12444 (13)	0.0212 (3)	
H15B	0.692879	0.525293	0.159060	0.025*	
C16B	0.78036 (18)	0.48170 (18)	0.02919 (15)	0.0285 (4)	
H16C	0.800752	0.422472	−0.008493	0.034*	
H16D	0.809921	0.553948	−0.001534	0.034*	
C17B	0.6827 (2)	0.22188 (18)	0.32701 (14)	0.0286 (4)	
H17B	0.764949	0.154736	0.327470	0.034*	
C18B	0.6080 (2)	0.2299 (2)	0.41801 (15)	0.0369 (5)	
H18C	0.524667	0.295177	0.422176	0.044*	
H18D	0.637505	0.170317	0.479470	0.044*	

### (RS)-Diethenyl{N-[(2-oxidonaphthalen-1-yl)methylidene]\ alaninato}silicon (2) Atomic displacement parameters (Å^2^)

**Table:**

	*U*^11^	*U*^22^	*U*^33^	*U*^12^	*U*^13^	*U*^23^
Si1A	0.01398 (19)	0.0189 (2)	0.0141 (2)	−0.00787 (16)	−0.00094 (15)	−0.00624 (16)
O1A	0.0164 (5)	0.0238 (6)	0.0149 (5)	−0.0115 (4)	−0.0014 (4)	−0.0045 (4)
O2A	0.0208 (6)	0.0358 (7)	0.0184 (6)	−0.0116 (5)	−0.0035 (5)	−0.0101 (5)
O3A	0.0352 (7)	0.0338 (7)	0.0195 (6)	−0.0125 (6)	−0.0088 (5)	−0.0106 (5)
N1A	0.0176 (6)	0.0246 (7)	0.0119 (6)	−0.0093 (5)	−0.0004 (5)	−0.0077 (5)
C1A	0.0142 (7)	0.0186 (7)	0.0128 (7)	−0.0073 (6)	−0.0017 (5)	−0.0044 (6)
C2A	0.0149 (7)	0.0160 (7)	0.0147 (7)	−0.0060 (5)	−0.0021 (5)	−0.0049 (6)
C3A	0.0215 (8)	0.0281 (8)	0.0118 (7)	−0.0137 (7)	0.0005 (6)	−0.0034 (6)
C4A	0.0253 (8)	0.0333 (9)	0.0121 (7)	−0.0143 (7)	−0.0033 (6)	−0.0053 (6)
C5A	0.0309 (9)	0.0534 (12)	0.0201 (8)	−0.0252 (9)	−0.0061 (7)	−0.0077 (8)
C6A	0.0340 (10)	0.0674 (15)	0.0290 (10)	−0.0349 (10)	−0.0052 (8)	−0.0116 (10)
C7A	0.0324 (10)	0.0623 (14)	0.0242 (9)	−0.0350 (10)	0.0016 (8)	−0.0089 (9)
C8A	0.0261 (9)	0.0439 (11)	0.0162 (8)	−0.0223 (8)	−0.0002 (7)	−0.0069 (7)
C9A	0.0162 (7)	0.0215 (7)	0.0153 (7)	−0.0086 (6)	−0.0018 (6)	−0.0058 (6)
C10A	0.0201 (8)	0.0282 (8)	0.0169 (8)	−0.0118 (7)	−0.0039 (6)	−0.0061 (6)
C11A	0.0158 (7)	0.0203 (7)	0.0148 (7)	−0.0077 (6)	−0.0003 (6)	−0.0057 (6)
C12A	0.0286 (9)	0.0408 (10)	0.0138 (7)	−0.0208 (8)	0.0021 (6)	−0.0118 (7)
C13A	0.0261 (8)	0.0211 (8)	0.0178 (8)	−0.0095 (6)	−0.0055 (6)	−0.0066 (6)
C14A	0.0233 (9)	0.0833 (18)	0.0489 (13)	−0.0165 (10)	0.0038 (9)	−0.0502 (13)
C15A	0.0216 (8)	0.0266 (8)	0.0261 (8)	−0.0130 (7)	−0.0001 (6)	−0.0121 (7)
C16A	0.0302 (9)	0.0244 (9)	0.0290 (9)	−0.0156 (7)	0.0012 (7)	−0.0071 (7)
C17A	0.0181 (7)	0.0203 (8)	0.0192 (8)	−0.0087 (6)	−0.0007 (6)	−0.0030 (6)
C18A	0.0302 (9)	0.0201 (8)	0.0254 (9)	−0.0076 (7)	0.0014 (7)	−0.0086 (7)
Si1B	0.0150 (2)	0.0196 (2)	0.0151 (2)	−0.00800 (16)	−0.00183 (15)	−0.00520 (16)
O1B	0.0170 (5)	0.0258 (6)	0.0262 (6)	−0.0108 (5)	0.0016 (4)	−0.0143 (5)
O2B	0.0158 (5)	0.0241 (6)	0.0249 (6)	−0.0039 (4)	−0.0054 (5)	−0.0089 (5)
O3B	0.0172 (6)	0.0282 (6)	0.0296 (7)	−0.0016 (5)	0.0004 (5)	−0.0142 (5)
N1B	0.0137 (6)	0.0186 (6)	0.0156 (6)	−0.0056 (5)	−0.0002 (5)	−0.0077 (5)
C1B	0.0144 (7)	0.0148 (7)	0.0142 (7)	−0.0050 (5)	−0.0011 (5)	−0.0041 (6)
C2B	0.0171 (7)	0.0168 (7)	0.0167 (7)	−0.0074 (6)	0.0004 (6)	−0.0050 (6)
C3B	0.0252 (8)	0.0223 (8)	0.0249 (8)	−0.0101 (7)	0.0016 (7)	−0.0134 (7)
C4B	0.0209 (8)	0.0198 (8)	0.0261 (9)	−0.0040 (6)	0.0029 (6)	−0.0124 (7)
C5B	0.0151 (7)	0.0297 (9)	0.0286 (9)	−0.0011 (7)	−0.0002 (6)	−0.0126 (7)
C6B	0.0129 (7)	0.0400 (10)	0.0306 (9)	−0.0058 (7)	−0.0025 (7)	−0.0130 (8)
C7B	0.0181 (8)	0.0367 (10)	0.0226 (8)	−0.0116 (7)	−0.0027 (6)	−0.0114 (7)
C8B	0.0163 (7)	0.0255 (8)	0.0154 (7)	−0.0070 (6)	−0.0018 (6)	−0.0071 (6)
C9B	0.0143 (7)	0.0162 (7)	0.0127 (7)	−0.0041 (5)	−0.0021 (5)	−0.0031 (5)
C10B	0.0157 (7)	0.0185 (7)	0.0192 (8)	−0.0029 (6)	−0.0006 (6)	−0.0058 (6)
C11B	0.0141 (7)	0.0162 (7)	0.0160 (7)	−0.0056 (6)	−0.0025 (5)	−0.0047 (6)
C12B	0.0162 (7)	0.0232 (8)	0.0190 (8)	−0.0058 (6)	0.0007 (6)	−0.0104 (6)
C13B	0.0169 (7)	0.0175 (7)	0.0174 (7)	−0.0050 (6)	−0.0015 (6)	−0.0034 (6)
C14B	0.0227 (8)	0.0383 (10)	0.0205 (8)	−0.0051 (7)	−0.0041 (7)	−0.0094 (7)
C15B	0.0188 (7)	0.0234 (8)	0.0242 (8)	−0.0098 (6)	−0.0041 (6)	−0.0063 (7)
C16B	0.0277 (9)	0.0301 (9)	0.0269 (9)	−0.0152 (8)	−0.0003 (7)	−0.0043 (7)
C17B	0.0351 (10)	0.0300 (9)	0.0235 (9)	−0.0148 (8)	−0.0093 (7)	−0.0026 (7)
C18B	0.0480 (12)	0.0443 (12)	0.0226 (9)	−0.0241 (10)	−0.0089 (8)	−0.0017 (8)

### (RS)-Diethenyl{N-[(2-oxidonaphthalen-1-yl)methylidene]\ alaninato}silicon (2) Geometric parameters (Å, º)

**Table:**

Si1A—O1A	1.7666 (12)	Si1B—O1B	1.7869 (12)
Si1A—O2A	1.8314 (12)	Si1B—O2B	1.8192 (12)
Si1A—C17A	1.8601 (17)	Si1B—N1B	1.8528 (14)
Si1A—N1A	1.8625 (14)	Si1B—C15B	1.8662 (17)
Si1A—C15A	1.8687 (17)	Si1B—C17B	1.8686 (19)
O1A—C2A	1.3181 (18)	O1B—C2B	1.3141 (19)
O2A—C13A	1.298 (2)	O2B—C13B	1.297 (2)
O3A—C13A	1.221 (2)	O3B—C13B	1.219 (2)
N1A—C11A	1.313 (2)	N1B—C11B	1.3115 (19)
N1A—C12A	1.480 (2)	N1B—C12B	1.4840 (19)
C1A—C2A	1.401 (2)	C1B—C2B	1.403 (2)
C1A—C11A	1.414 (2)	C1B—C11B	1.418 (2)
C1A—C9A	1.448 (2)	C1B—C9B	1.452 (2)
C2A—C3A	1.415 (2)	C2B—C3B	1.423 (2)
C3A—C4A	1.363 (2)	C3B—C4B	1.364 (2)
C3A—H3A	0.9500	C3B—H3B	0.9500
C4A—C10A	1.425 (2)	C4B—C10B	1.427 (2)
C4A—H4A	0.9500	C4B—H4B	0.9500
C5A—C6A	1.370 (3)	C5B—C6B	1.372 (3)
C5A—C10A	1.414 (2)	C5B—C10B	1.415 (2)
C5A—H5A	0.9500	C5B—H5B	0.9500
C6A—C7A	1.399 (3)	C6B—C7B	1.400 (3)
C6A—H6A	0.9500	C6B—H6B	0.9500
C7A—C8A	1.377 (2)	C7B—C8B	1.381 (2)
C7A—H7A	0.9500	C7B—H7B	0.9500
C8A—C9A	1.415 (2)	C8B—C9B	1.413 (2)
C8A—H8A	0.9500	C8B—H8B	0.9500
C9A—C10A	1.417 (2)	C9B—C10B	1.417 (2)
C11A—H11A	0.97 (2)	C11B—H11B	0.979 (19)
C12A—C14A	1.507 (3)	C12B—C14B	1.513 (2)
C12A—C13A	1.518 (2)	C12B—C13B	1.519 (2)
C12A—H12A	1.04 (2)	C12B—H12B	1.04 (2)
C14A—H14A	0.9800	C14B—H14D	0.9800
C14A—H14B	0.9800	C14B—H14E	0.9800
C14A—H14C	0.9800	C14B—H14F	0.9800
C15A—C16A	1.325 (3)	C15B—C16B	1.323 (2)
C15A—H15A	0.9500	C15B—H15B	0.9500
C16A—H16A	0.9500	C16B—H16C	0.9500
C16A—H16B	0.9500	C16B—H16D	0.9500
C17A—C18A	1.331 (2)	C17B—C18B	1.317 (3)
C17A—H17A	0.9500	C17B—H17B	0.9500
C18A—H18A	0.9500	C18B—H18C	0.9500
C18A—H18B	0.9500	C18B—H18D	0.9500
			
O1A—Si1A—O2A	169.71 (6)	O1B—Si1B—O2B	172.71 (6)
O1A—Si1A—C17A	96.47 (7)	O1B—Si1B—N1B	89.42 (6)
O2A—Si1A—C17A	92.55 (7)	O2B—Si1B—N1B	83.49 (6)
O1A—Si1A—N1A	89.15 (6)	O1B—Si1B—C15B	89.31 (7)
O2A—Si1A—N1A	82.74 (6)	O2B—Si1B—C15B	92.61 (7)
C17A—Si1A—N1A	113.14 (7)	N1B—Si1B—C15B	118.46 (7)
O1A—Si1A—C15A	91.69 (7)	O1B—Si1B—C17B	94.97 (8)
O2A—Si1A—C15A	88.27 (7)	O2B—Si1B—C17B	89.93 (8)
C17A—Si1A—C15A	118.83 (7)	N1B—Si1B—C17B	118.97 (7)
N1A—Si1A—C15A	127.55 (7)	C15B—Si1B—C17B	122.43 (8)
C2A—O1A—Si1A	131.74 (10)	C2B—O1B—Si1B	132.29 (10)
C13A—O2A—Si1A	120.54 (11)	C13B—O2B—Si1B	120.35 (10)
C11A—N1A—C12A	116.11 (13)	C11B—N1B—C12B	116.35 (13)
C11A—N1A—Si1A	127.02 (11)	C11B—N1B—Si1B	127.51 (11)
C12A—N1A—Si1A	116.48 (10)	C12B—N1B—Si1B	115.75 (10)
C2A—C1A—C11A	118.41 (14)	C2B—C1B—C11B	118.61 (14)
C2A—C1A—C9A	120.12 (14)	C2B—C1B—C9B	120.28 (14)
C11A—C1A—C9A	121.18 (14)	C11B—C1B—C9B	120.89 (14)
O1A—C2A—C1A	121.93 (14)	O1B—C2B—C1B	121.75 (14)
O1A—C2A—C3A	117.64 (13)	O1B—C2B—C3B	118.16 (14)
C1A—C2A—C3A	120.36 (14)	C1B—C2B—C3B	120.03 (14)
C4A—C3A—C2A	119.93 (14)	C4B—C3B—C2B	119.80 (15)
C4A—C3A—H3A	120.0	C4B—C3B—H3B	120.1
C2A—C3A—H3A	120.0	C2B—C3B—H3B	120.1
C3A—C4A—C10A	121.83 (15)	C3B—C4B—C10B	122.20 (15)
C3A—C4A—H4A	119.1	C3B—C4B—H4B	118.9
C10A—C4A—H4A	119.1	C10B—C4B—H4B	118.9
C6A—C5A—C10A	120.47 (17)	C6B—C5B—C10B	120.87 (16)
C6A—C5A—H5A	119.8	C6B—C5B—H5B	119.6
C10A—C5A—H5A	119.8	C10B—C5B—H5B	119.6
C5A—C6A—C7A	120.01 (17)	C5B—C6B—C7B	119.77 (16)
C5A—C6A—H6A	120.0	C5B—C6B—H6B	120.1
C7A—C6A—H6A	120.0	C7B—C6B—H6B	120.1
C8A—C7A—C6A	120.93 (17)	C8B—C7B—C6B	120.76 (16)
C8A—C7A—H7A	119.5	C8B—C7B—H7B	119.6
C6A—C7A—H7A	119.5	C6B—C7B—H7B	119.6
C7A—C8A—C9A	120.46 (16)	C7B—C8B—C9B	120.62 (15)
C7A—C8A—H8A	119.8	C7B—C8B—H8B	119.7
C9A—C8A—H8A	119.8	C9B—C8B—H8B	119.7
C8A—C9A—C10A	118.33 (14)	C8B—C9B—C10B	118.46 (14)
C8A—C9A—C1A	123.49 (14)	C8B—C9B—C1B	123.24 (14)
C10A—C9A—C1A	118.18 (14)	C10B—C9B—C1B	118.28 (14)
C5A—C10A—C9A	119.78 (15)	C5B—C10B—C9B	119.49 (15)
C5A—C10A—C4A	120.66 (15)	C5B—C10B—C4B	121.24 (15)
C9A—C10A—C4A	119.56 (14)	C9B—C10B—C4B	119.26 (14)
N1A—C11A—C1A	124.89 (14)	N1B—C11B—C1B	125.08 (14)
N1A—C11A—H11A	116.1 (12)	N1B—C11B—H11B	116.3 (11)
C1A—C11A—H11A	119.0 (12)	C1B—C11B—H11B	118.5 (11)
N1A—C12A—C14A	111.27 (16)	N1B—C12B—C14B	112.29 (14)
N1A—C12A—C13A	105.57 (13)	N1B—C12B—C13B	105.41 (13)
C14A—C12A—C13A	113.00 (15)	C14B—C12B—C13B	110.99 (13)
N1A—C12A—H12A	110.2 (13)	N1B—C12B—H12B	110.9 (12)
C14A—C12A—H12A	107.6 (13)	C14B—C12B—H12B	106.0 (12)
C13A—C12A—H12A	109.3 (13)	C13B—C12B—H12B	111.4 (12)
O3A—C13A—O2A	124.93 (16)	O3B—C13B—O2B	124.76 (15)
O3A—C13A—C12A	121.72 (15)	O3B—C13B—C12B	121.68 (15)
O2A—C13A—C12A	113.35 (14)	O2B—C13B—C12B	113.55 (13)
C12A—C14A—H14A	109.5	C12B—C14B—H14D	109.5
C12A—C14A—H14B	109.5	C12B—C14B—H14E	109.5
H14A—C14A—H14B	109.5	H14D—C14B—H14E	109.5
C12A—C14A—H14C	109.5	C12B—C14B—H14F	109.5
H14A—C14A—H14C	109.5	H14D—C14B—H14F	109.5
H14B—C14A—H14C	109.5	H14E—C14B—H14F	109.5
C16A—C15A—Si1A	127.71 (14)	C16B—C15B—Si1B	126.03 (14)
C16A—C15A—H15A	116.1	C16B—C15B—H15B	117.0
Si1A—C15A—H15A	116.1	Si1B—C15B—H15B	117.0
C15A—C16A—H16A	120.0	C15B—C16B—H16C	120.0
C15A—C16A—H16B	120.0	C15B—C16B—H16D	120.0
H16A—C16A—H16B	120.0	H16C—C16B—H16D	120.0
C18A—C17A—Si1A	125.48 (14)	C18B—C17B—Si1B	127.23 (17)
C18A—C17A—H17A	117.3	C18B—C17B—H17B	116.4
Si1A—C17A—H17A	117.3	Si1B—C17B—H17B	116.4
C17A—C18A—H18A	120.0	C17B—C18B—H18C	120.0
C17A—C18A—H18B	120.0	C17B—C18B—H18D	120.0
H18A—C18A—H18B	120.0	H18C—C18B—H18D	120.0
			
O2A—Si1A—O1A—C2A	66.8 (4)	C15A—Si1A—C17A—C18A	92.75 (17)
C17A—Si1A—O1A—C2A	−84.24 (14)	N1B—Si1B—O1B—C2B	24.90 (15)
N1A—Si1A—O1A—C2A	28.95 (14)	C15B—Si1B—O1B—C2B	143.37 (15)
C15A—Si1A—O1A—C2A	156.50 (14)	C17B—Si1B—O1B—C2B	−94.14 (16)
O1A—Si1A—O2A—C13A	−26.9 (4)	N1B—Si1B—O2B—C13B	9.77 (12)
C17A—Si1A—O2A—C13A	124.37 (13)	C15B—Si1B—O2B—C13B	−108.58 (13)
N1A—Si1A—O2A—C13A	11.36 (13)	C17B—Si1B—O2B—C13B	128.96 (13)
C15A—Si1A—O2A—C13A	−116.84 (14)	O1B—Si1B—N1B—C11B	−20.45 (14)
O1A—Si1A—N1A—C11A	−22.79 (14)	O2B—Si1B—N1B—C11B	161.21 (14)
O2A—Si1A—N1A—C11A	163.56 (15)	C15B—Si1B—N1B—C11B	−109.35 (14)
C17A—Si1A—N1A—C11A	73.88 (16)	C17B—Si1B—N1B—C11B	74.91 (16)
C15A—Si1A—N1A—C11A	−114.28 (15)	O1B—Si1B—N1B—C12B	167.04 (11)
O1A—Si1A—N1A—C12A	164.62 (12)	O2B—Si1B—N1B—C12B	−11.31 (11)
O2A—Si1A—N1A—C12A	−9.03 (12)	C15B—Si1B—N1B—C12B	78.14 (13)
C17A—Si1A—N1A—C12A	−98.71 (13)	C17B—Si1B—N1B—C12B	−97.60 (13)
C15A—Si1A—N1A—C12A	73.13 (15)	Si1B—O1B—C2B—C1B	−15.6 (2)
Si1A—O1A—C2A—C1A	−20.1 (2)	Si1B—O1B—C2B—C3B	167.16 (12)
Si1A—O1A—C2A—C3A	162.85 (12)	C11B—C1B—C2B—O1B	−6.3 (2)
C11A—C1A—C2A—O1A	−4.5 (2)	C9B—C1B—C2B—O1B	179.00 (14)
C9A—C1A—C2A—O1A	−178.41 (14)	C11B—C1B—C2B—C3B	170.92 (15)
C11A—C1A—C2A—C3A	172.47 (15)	C9B—C1B—C2B—C3B	−3.8 (2)
C9A—C1A—C2A—C3A	−1.4 (2)	O1B—C2B—C3B—C4B	178.21 (16)
O1A—C2A—C3A—C4A	177.79 (15)	C1B—C2B—C3B—C4B	0.9 (3)
C1A—C2A—C3A—C4A	0.7 (2)	C2B—C3B—C4B—C10B	1.5 (3)
C2A—C3A—C4A—C10A	−0.4 (3)	C10B—C5B—C6B—C7B	0.2 (3)
C10A—C5A—C6A—C7A	0.1 (4)	C5B—C6B—C7B—C8B	−0.5 (3)
C5A—C6A—C7A—C8A	−0.4 (4)	C6B—C7B—C8B—C9B	−0.3 (3)
C6A—C7A—C8A—C9A	−0.5 (3)	C7B—C8B—C9B—C10B	1.5 (2)
C7A—C8A—C9A—C10A	1.7 (3)	C7B—C8B—C9B—C1B	−179.90 (15)
C7A—C8A—C9A—C1A	−177.87 (18)	C2B—C1B—C9B—C8B	−174.36 (15)
C2A—C1A—C9A—C8A	−178.60 (16)	C11B—C1B—C9B—C8B	11.0 (2)
C11A—C1A—C9A—C8A	7.7 (3)	C2B—C1B—C9B—C10B	4.3 (2)
C2A—C1A—C9A—C10A	1.8 (2)	C11B—C1B—C9B—C10B	−170.35 (14)
C11A—C1A—C9A—C10A	−171.89 (15)	C6B—C5B—C10B—C9B	1.0 (3)
C6A—C5A—C10A—C9A	1.1 (3)	C6B—C5B—C10B—C4B	−177.58 (17)
C6A—C5A—C10A—C4A	−179.8 (2)	C8B—C9B—C10B—C5B	−1.8 (2)
C8A—C9A—C10A—C5A	−2.0 (3)	C1B—C9B—C10B—C5B	179.49 (15)
C1A—C9A—C10A—C5A	177.61 (17)	C8B—C9B—C10B—C4B	176.80 (15)
C8A—C9A—C10A—C4A	178.90 (17)	C1B—C9B—C10B—C4B	−1.9 (2)
C1A—C9A—C10A—C4A	−1.5 (2)	C3B—C4B—C10B—C5B	177.63 (17)
C3A—C4A—C10A—C5A	−178.30 (18)	C3B—C4B—C10B—C9B	−1.0 (3)
C3A—C4A—C10A—C9A	0.8 (3)	C12B—N1B—C11B—C1B	−179.07 (14)
C12A—N1A—C11A—C1A	−177.86 (15)	Si1B—N1B—C11B—C1B	8.5 (2)
Si1A—N1A—C11A—C1A	9.5 (2)	C2B—C1B—C11B—N1B	9.1 (2)
C2A—C1A—C11A—N1A	8.6 (2)	C9B—C1B—C11B—N1B	−176.22 (15)
C9A—C1A—C11A—N1A	−177.52 (15)	C11B—N1B—C12B—C14B	76.33 (18)
C11A—N1A—C12A—C14A	69.5 (2)	Si1B—N1B—C12B—C14B	−110.29 (14)
Si1A—N1A—C12A—C14A	−117.10 (15)	C11B—N1B—C12B—C13B	−162.71 (13)
C11A—N1A—C12A—C13A	−167.58 (14)	Si1B—N1B—C12B—C13B	10.67 (16)
Si1A—N1A—C12A—C13A	5.83 (18)	Si1B—O2B—C13B—O3B	173.82 (13)
Si1A—O2A—C13A—O3A	169.53 (14)	Si1B—O2B—C13B—C12B	−5.67 (18)
Si1A—O2A—C13A—C12A	−10.61 (19)	N1B—C12B—C13B—O3B	177.16 (15)
N1A—C12A—C13A—O3A	−177.53 (16)	C14B—C12B—C13B—O3B	−61.0 (2)
C14A—C12A—C13A—O3A	−55.7 (2)	N1B—C12B—C13B—O2B	−3.33 (18)
N1A—C12A—C13A—O2A	2.6 (2)	C14B—C12B—C13B—O2B	118.49 (16)
C14A—C12A—C13A—O2A	124.42 (18)	O1B—Si1B—C15B—C16B	−139.93 (17)
O1A—Si1A—C15A—C16A	−6.77 (17)	O2B—Si1B—C15B—C16B	33.04 (17)
O2A—Si1A—C15A—C16A	162.93 (17)	N1B—Si1B—C15B—C16B	−50.97 (18)
C17A—Si1A—C15A—C16A	−105.10 (17)	C17B—Si1B—C15B—C16B	124.62 (17)
N1A—Si1A—C15A—C16A	83.47 (18)	O1B—Si1B—C17B—C18B	5.56 (19)
O1A—Si1A—C17A—C18A	−2.78 (16)	O2B—Si1B—C17B—C18B	−169.05 (19)
O2A—Si1A—C17A—C18A	−177.82 (15)	N1B—Si1B—C17B—C18B	−86.5 (2)
N1A—Si1A—C17A—C18A	−94.63 (16)	C15B—Si1B—C17B—C18B	97.9 (2)

### (RS)-Diethenyl{N-[(2-oxidonaphthalen-1-yl)methylidene]\ alaninato}silicon (2) Hydrogen-bond geometry (Å, º)

*Cg*3 and *Cg*14 are the centroids of the C1*A*–C4*A*,C9*A*,C10*A* and C5*B*–C10*B* rings, respectively.

**Table:**

*D*—H···*A*	*D*—H	H···*A*	*D*···*A*	*D*—H···*A*
C8*A*—H8*A*···O3*B*^i^	0.95	2.56	3.429 (2)	152
C11*A*—H11*A*···O3*B*^i^	0.97 (2)	2.56 (2)	3.526 (2)	172.3 (17)
C12*A*—H12*A*···O3*B*^ii^	1.04 (2)	2.53 (2)	3.370 (2)	138.0 (18)
C16*A*—H16*A*···O1*A*	0.95	2.39	2.829 (2)	108
C18*A*—H18*A*···O1*A*	0.95	2.45	2.911 (2)	110
C8*B*—H8*B*···O3*A*^iii^	0.95	2.56	3.479 (2)	163
C11*B*—H11*B*···O3*A*^iii^	0.979 (19)	2.30 (2)	3.260 (2)	165.7 (15)
C16*B*—H16*C*···O2*B*	0.95	2.58	2.937 (2)	103
C18*B*—H18*C*···O1*B*	0.95	2.46	2.917 (2)	109
C4*A*—H4*A*···*Cg*14	0.95	2.63	3.3641 (18)	134
C4*B*—H4*B*···*Cg*3^iv^	0.95	2.90	3.744 (2)	149

Symmetry codes: (i) −*x*+2, −*y*+1, −*z*; (ii) *x*, *y*+1, *z*; (iii) −*x*+1, −*y*+1, −*z*; (iv) −*x*, −*y*+1, −*z*+1.

### (RS)-Methyl{N-[(2-oxidonaphthalen-1-yl)methylidene]alaninato}phenylsilicon chloroform hemisolvate (3) Crystal data

**Table:**

2C_21_H_19_NO_3_Si·CHCl_3_	*D*_x_ = 1.356 Mg m^−^^3^
*M**_r_* = 842.29	Melting point: 392 K
Monoclinic, *I*2/*a*	Mo *K*α radiation, λ = 0.71073 Å
*a* = 10.7122 (5) Å	Cell parameters from 29869 reflections
*b* = 27.2203 (13) Å	θ = 2.7–29.5°
*c* = 14.1716 (8) Å	µ = 0.33 mm^−^^1^
β = 93.546 (4)°	*T* = 153 K
*V* = 4124.4 (4) Å^3^	Prism, yellow
*Z* = 4	0.40 × 0.35 × 0.30 mm
*F*(000) = 1752	

### (RS)-Methyl{N-[(2-oxidonaphthalen-1-yl)methylidene]alaninato}phenylsilicon chloroform hemisolvate (3) Data collection

**Table:**

Stoe IPDS 2T diffractometer	4732 independent reflections
Radiation source: sealed X-ray tube, 12 x 0.4 mm long-fine focus	3956 reflections with *I* > 2σ(*I*)
Plane graphite monochromator	*R*_int_ = 0.027
Detector resolution: 6.67 pixels mm^-1^	θ_max_ = 27.5°, θ_min_ = 2.7°
rotation method, ω scans	*h* = −11→13
Absorption correction: integration (X-RED; Stoe, 2024)	*k* = −35→35
*T*_min_ = 0.733, *T*_max_ = 0.773	*l* = −18→18
25032 measured reflections	

### (RS)-Methyl{N-[(2-oxidonaphthalen-1-yl)methylidene]alaninato}phenylsilicon chloroform hemisolvate (3) Refinement

**Table:**

Refinement on *F*^2^	Primary atom site location: structure-invariant direct methods
Least-squares matrix: full	Hydrogen site location: inferred from neighbouring sites
*R*[*F*^2^ > 2σ(*F*^2^)] = 0.044	H-atom parameters constrained
*wR*(*F*^2^) = 0.118	*w* = 1/[σ^2^(*F*_o_^2^) + (0.0536*P*)^2^ + 5.2844*P*] where *P* = (*F*_o_^2^ + 2*F*_c_^2^)/3
*S* = 1.06	(Δ/σ)_max_ < 0.001
4732 reflections	Δρ_max_ = 0.53 e Å^−^^3^
273 parameters	Δρ_min_ = −0.47 e Å^−^^3^
27 restraints	

### (RS)-Methyl{N-[(2-oxidonaphthalen-1-yl)methylidene]alaninato}phenylsilicon chloroform hemisolvate (3) Special details

**Table:**

Geometry. All esds (except the esd in the dihedral angle between two l.s. planes) are estimated using the full covariance matrix. The cell esds are taken into account individually in the estimation of esds in distances, angles and torsion angles; correlations between esds in cell parameters are only used when they are defined by crystal symmetry. An approximate (isotropic) treatment of cell esds is used for estimating esds involving l.s. planes.

### (RS)-Methyl{N-[(2-oxidonaphthalen-1-yl)methylidene]alaninato}phenylsilicon chloroform hemisolvate (3) Fractional atomic coordinates and isotropic or equivalent isotropic displacement parameters (Å^2^)

**Table:**

	*x*	*y*	*z*	*U*_iso_*/*U*_eq_	Occ. (<1)
Si1	0.73346 (4)	0.11367 (2)	0.24625 (3)	0.02389 (12)	
O1	0.80416 (11)	0.17243 (4)	0.23133 (9)	0.0276 (3)	
O2	0.68432 (12)	0.04854 (5)	0.25171 (11)	0.0362 (3)	
O3	0.72151 (14)	−0.02779 (5)	0.20106 (14)	0.0518 (4)	
N1	0.88541 (12)	0.08514 (5)	0.22233 (10)	0.0227 (3)	
C1	1.01609 (15)	0.15306 (6)	0.27696 (11)	0.0235 (3)	
C2	0.91653 (15)	0.18658 (6)	0.26497 (12)	0.0247 (3)	
C3	0.93682 (18)	0.23727 (7)	0.28507 (14)	0.0342 (4)	
H3	0.870908	0.260260	0.273547	0.041*	
C4	1.05079 (19)	0.25269 (7)	0.32080 (15)	0.0367 (4)	
H4	1.062691	0.286613	0.334294	0.044*	
C5	1.26924 (18)	0.23662 (8)	0.38000 (15)	0.0396 (5)	
H5	1.279329	0.270410	0.395457	0.047*	
C6	1.36669 (19)	0.20500 (9)	0.39782 (17)	0.0455 (5)	
H6	1.443728	0.216582	0.426328	0.055*	
C7	1.35227 (19)	0.15544 (9)	0.3738 (2)	0.0526 (6)	
H7	1.420456	0.133517	0.385613	0.063*	
C8	1.24070 (18)	0.13776 (8)	0.33317 (18)	0.0434 (5)	
H8	1.233219	0.103974	0.317011	0.052*	
C9	1.13767 (16)	0.16947 (6)	0.31545 (12)	0.0272 (3)	
C10	1.15333 (16)	0.21978 (7)	0.33881 (13)	0.0297 (4)	
C11	0.99556 (15)	0.10408 (6)	0.24502 (12)	0.0245 (3)	
H11	1.066696	0.083647	0.239747	0.029*	
C12	0.87957 (16)	0.03390 (6)	0.18807 (13)	0.0277 (4)	
H12	0.946827	0.014308	0.222646	0.033*	
C13	0.75250 (17)	0.01485 (7)	0.21394 (15)	0.0342 (4)	
C14	0.8971 (2)	0.03134 (7)	0.08268 (16)	0.0439 (5)	
H14A	0.838706	0.053990	0.049184	0.066*	
H14B	0.880891	−0.002225	0.060130	0.066*	
H14C	0.983133	0.040607	0.070721	0.066*	
C15	0.7010 (2)	0.12520 (11)	0.37195 (14)	0.0488 (6)	
H15A	0.779736	0.124746	0.411032	0.073*	
H15B	0.645288	0.099560	0.393644	0.073*	
H15C	0.660911	0.157353	0.377317	0.073*	
C16	0.61023 (15)	0.12617 (6)	0.14837 (11)	0.0228 (3)	
C17	0.49279 (16)	0.10288 (6)	0.14733 (13)	0.0274 (3)	
H17	0.475797	0.080901	0.197001	0.033*	
C18	0.40088 (16)	0.11129 (6)	0.07522 (14)	0.0306 (4)	
H18	0.321600	0.095794	0.077030	0.037*	
C19	0.42505 (17)	0.14224 (6)	0.00083 (13)	0.0304 (4)	
H19	0.363385	0.147240	−0.049346	0.036*	
C20	0.53985 (17)	0.16589 (7)	0.00003 (12)	0.0301 (4)	
H20	0.556927	0.187107	−0.050810	0.036*	
C21	0.63015 (15)	0.15847 (6)	0.07400 (12)	0.0259 (3)	
H21	0.707108	0.175809	0.073811	0.031*	
C22	0.0431 (5)	−0.00264 (17)	0.4526 (4)	0.0521 (11)	0.5
H22	0.093644	−0.015674	0.401125	0.063*	0.5
Cl1	0.06967 (19)	0.05991 (6)	0.46264 (13)	0.0800 (5)	0.5
Cl2	−0.11495 (19)	−0.01296 (9)	0.42105 (14)	0.0974 (6)	0.5
Cl3	0.0861 (3)	−0.03383 (9)	0.55587 (12)	0.1154 (9)	0.5

### (RS)-Methyl{N-[(2-oxidonaphthalen-1-yl)methylidene]alaninato}phenylsilicon chloroform hemisolvate (3) Atomic displacement parameters (Å^2^)

**Table:**

	*U*^11^	*U*^22^	*U*^33^	*U*^12^	*U*^13^	*U*^23^
Si1	0.0171 (2)	0.0298 (2)	0.0245 (2)	0.00547 (17)	−0.00076 (16)	0.00147 (17)
O1	0.0219 (6)	0.0254 (6)	0.0344 (6)	0.0060 (4)	−0.0079 (5)	−0.0069 (5)
O2	0.0197 (6)	0.0336 (7)	0.0555 (8)	0.0033 (5)	0.0039 (5)	0.0183 (6)
O3	0.0332 (8)	0.0273 (7)	0.0945 (13)	−0.0086 (6)	0.0019 (8)	0.0107 (7)
N1	0.0192 (6)	0.0199 (6)	0.0289 (7)	0.0020 (5)	0.0009 (5)	0.0015 (5)
C1	0.0200 (7)	0.0243 (7)	0.0262 (7)	0.0025 (6)	0.0008 (6)	−0.0020 (6)
C2	0.0237 (8)	0.0251 (8)	0.0250 (7)	0.0031 (6)	−0.0019 (6)	−0.0057 (6)
C3	0.0307 (9)	0.0263 (8)	0.0450 (11)	0.0068 (7)	−0.0041 (8)	−0.0099 (7)
C4	0.0354 (10)	0.0276 (9)	0.0468 (11)	0.0003 (7)	−0.0006 (8)	−0.0149 (8)
C5	0.0305 (10)	0.0422 (10)	0.0459 (11)	−0.0083 (8)	0.0017 (8)	−0.0155 (9)
C6	0.0228 (9)	0.0578 (13)	0.0551 (13)	−0.0084 (9)	−0.0038 (8)	−0.0130 (10)
C7	0.0203 (9)	0.0506 (13)	0.0854 (18)	0.0035 (9)	−0.0084 (10)	−0.0074 (12)
C8	0.0222 (9)	0.0348 (10)	0.0721 (15)	0.0019 (7)	−0.0067 (9)	−0.0081 (10)
C9	0.0203 (8)	0.0305 (8)	0.0310 (8)	0.0001 (6)	0.0023 (6)	−0.0043 (7)
C10	0.0243 (8)	0.0339 (9)	0.0312 (8)	−0.0024 (7)	0.0031 (7)	−0.0088 (7)
C11	0.0188 (7)	0.0241 (8)	0.0307 (8)	0.0034 (6)	0.0023 (6)	0.0009 (6)
C12	0.0226 (8)	0.0176 (7)	0.0426 (10)	0.0000 (6)	0.0002 (7)	0.0017 (7)
C13	0.0214 (8)	0.0288 (9)	0.0518 (11)	−0.0004 (7)	−0.0020 (8)	0.0132 (8)
C14	0.0608 (14)	0.0269 (9)	0.0448 (11)	−0.0109 (9)	0.0106 (10)	−0.0095 (8)
C15	0.0287 (10)	0.0892 (18)	0.0285 (9)	0.0089 (11)	0.0022 (8)	−0.0006 (10)
C16	0.0200 (7)	0.0221 (7)	0.0260 (7)	0.0052 (6)	−0.0023 (6)	−0.0036 (6)
C17	0.0238 (8)	0.0214 (7)	0.0366 (9)	0.0015 (6)	−0.0023 (7)	0.0026 (6)
C18	0.0223 (8)	0.0231 (8)	0.0452 (10)	−0.0006 (6)	−0.0073 (7)	−0.0013 (7)
C19	0.0257 (8)	0.0302 (9)	0.0338 (9)	0.0046 (7)	−0.0098 (7)	−0.0041 (7)
C20	0.0294 (9)	0.0342 (9)	0.0263 (8)	0.0028 (7)	−0.0010 (7)	0.0024 (7)
C21	0.0205 (7)	0.0300 (8)	0.0270 (8)	0.0008 (6)	0.0003 (6)	−0.0026 (6)
C22	0.051 (3)	0.048 (2)	0.057 (3)	0.013 (2)	−0.002 (2)	−0.004 (2)
Cl1	0.1004 (13)	0.0588 (8)	0.0810 (10)	−0.0176 (8)	0.0073 (9)	−0.0064 (7)
Cl2	0.0786 (12)	0.1280 (17)	0.0829 (12)	−0.0401 (11)	−0.0170 (9)	0.0105 (11)
Cl3	0.183 (2)	0.1131 (15)	0.0481 (8)	0.0769 (16)	−0.0128 (11)	0.0231 (9)

### (RS)-Methyl{N-[(2-oxidonaphthalen-1-yl)methylidene]alaninato}phenylsilicon chloroform hemisolvate (3) Geometric parameters (Å, º)

**Table:**

Si1—O1	1.7879 (13)	C9—C10	1.416 (2)
Si1—O2	1.8522 (14)	C11—H11	0.9500
Si1—N1	1.8534 (14)	C12—C14	1.518 (3)
Si1—C15	1.863 (2)	C12—C13	1.522 (2)
Si1—C16	1.8857 (16)	C12—H12	1.0000
O1—C2	1.324 (2)	C14—H14A	0.9800
O2—C13	1.308 (2)	C14—H14B	0.9800
O3—C13	1.218 (2)	C14—H14C	0.9800
N1—C11	1.309 (2)	C15—H15A	0.9800
N1—C12	1.477 (2)	C15—H15B	0.9800
C1—C2	1.406 (2)	C15—H15C	0.9800
C1—C11	1.421 (2)	C16—C21	1.399 (2)
C1—C9	1.451 (2)	C16—C17	1.408 (2)
C2—C3	1.423 (2)	C17—C18	1.393 (2)
C3—C4	1.359 (3)	C17—H17	0.9500
C3—H3	0.9500	C18—C19	1.386 (3)
C4—C10	1.428 (3)	C18—H18	0.9500
C4—H4	0.9500	C19—C20	1.389 (3)
C5—C6	1.364 (3)	C19—H19	0.9500
C5—C10	1.415 (3)	C20—C21	1.396 (2)
C5—H5	0.9500	C20—H20	0.9500
C6—C7	1.398 (3)	C21—H21	0.9500
C6—H6	0.9500	C22—Cl3	1.729 (5)
C7—C8	1.381 (3)	C22—Cl1	1.730 (5)
C7—H7	0.9500	C22—Cl2	1.747 (5)
C8—C9	1.411 (3)	C22—H22	1.0000
C8—H8	0.9500		
			
O1—Si1—O2	169.99 (6)	C1—C11—H11	117.7
O1—Si1—N1	88.28 (6)	N1—C12—C14	111.19 (14)
O2—Si1—N1	82.06 (6)	N1—C12—C13	105.24 (14)
O1—Si1—C15	93.89 (10)	C14—C12—C13	112.84 (16)
O2—Si1—C15	92.92 (10)	N1—C12—H12	109.2
N1—Si1—C15	117.61 (8)	C14—C12—H12	109.2
O1—Si1—C16	91.95 (6)	C13—C12—H12	109.2
O2—Si1—C16	90.90 (7)	O3—C13—O2	125.16 (18)
N1—Si1—C16	121.59 (7)	O3—C13—C12	121.88 (18)
C15—Si1—C16	120.63 (8)	O2—C13—C12	112.95 (15)
C2—O1—Si1	126.87 (11)	C12—C14—H14A	109.5
C13—O2—Si1	119.13 (11)	C12—C14—H14B	109.5
C11—N1—C12	118.14 (13)	H14A—C14—H14B	109.5
C11—N1—Si1	125.34 (11)	C12—C14—H14C	109.5
C12—N1—Si1	115.84 (10)	H14A—C14—H14C	109.5
C2—C1—C11	117.99 (15)	H14B—C14—H14C	109.5
C2—C1—C9	120.14 (15)	Si1—C15—H15A	109.5
C11—C1—C9	121.76 (14)	Si1—C15—H15B	109.5
O1—C2—C1	121.36 (14)	H15A—C15—H15B	109.5
O1—C2—C3	118.58 (15)	Si1—C15—H15C	109.5
C1—C2—C3	119.99 (15)	H15A—C15—H15C	109.5
C4—C3—C2	119.78 (17)	H15B—C15—H15C	109.5
C4—C3—H3	120.1	C21—C16—C17	116.93 (15)
C2—C3—H3	120.1	C21—C16—Si1	122.35 (12)
C3—C4—C10	122.42 (17)	C17—C16—Si1	120.72 (13)
C3—C4—H4	118.8	C18—C17—C16	121.61 (16)
C10—C4—H4	118.8	C18—C17—H17	119.2
C6—C5—C10	120.90 (19)	C16—C17—H17	119.2
C6—C5—H5	119.6	C19—C18—C17	120.05 (16)
C10—C5—H5	119.6	C19—C18—H18	120.0
C5—C6—C7	119.48 (18)	C17—C18—H18	120.0
C5—C6—H6	120.3	C18—C19—C20	119.67 (16)
C7—C6—H6	120.3	C18—C19—H19	120.2
C8—C7—C6	121.2 (2)	C20—C19—H19	120.2
C8—C7—H7	119.4	C19—C20—C21	119.97 (16)
C6—C7—H7	119.4	C19—C20—H20	120.0
C7—C8—C9	120.55 (19)	C21—C20—H20	120.0
C7—C8—H8	119.7	C20—C21—C16	121.70 (16)
C9—C8—H8	119.7	C20—C21—H21	119.1
C8—C9—C10	118.02 (16)	C16—C21—H21	119.1
C8—C9—C1	123.53 (16)	Cl3—C22—Cl1	112.3 (3)
C10—C9—C1	118.43 (15)	Cl3—C22—Cl2	109.9 (3)
C5—C10—C9	119.86 (17)	Cl1—C22—Cl2	109.3 (3)
C5—C10—C4	121.06 (17)	Cl3—C22—H22	108.4
C9—C10—C4	119.08 (16)	Cl1—C22—H22	108.4
N1—C11—C1	124.54 (15)	Cl2—C22—H22	108.4
N1—C11—H11	117.7		
			
O2—Si1—O1—C2	−58.3 (4)	C8—C9—C10—C5	−1.0 (3)
N1—Si1—O1—C2	−43.17 (14)	C1—C9—C10—C5	177.48 (17)
C15—Si1—O1—C2	74.39 (15)	C8—C9—C10—C4	179.46 (19)
C16—Si1—O1—C2	−164.73 (14)	C1—C9—C10—C4	−2.1 (3)
O1—Si1—O2—C13	−3.2 (5)	C3—C4—C10—C5	−177.1 (2)
N1—Si1—O2—C13	−18.47 (14)	C3—C4—C10—C9	2.4 (3)
C15—Si1—O2—C13	−135.95 (15)	C12—N1—C11—C1	178.57 (15)
C16—Si1—O2—C13	103.32 (15)	Si1—N1—C11—C1	−11.2 (2)
O1—Si1—N1—C11	32.50 (14)	C2—C1—C11—N1	−14.1 (3)
O2—Si1—N1—C11	−150.13 (15)	C9—C1—C11—N1	169.82 (16)
C15—Si1—N1—C11	−60.99 (18)	C11—N1—C12—C14	−84.7 (2)
C16—Si1—N1—C11	123.72 (14)	Si1—N1—C12—C14	104.15 (16)
O1—Si1—N1—C12	−157.11 (12)	C11—N1—C12—C13	152.78 (15)
O2—Si1—N1—C12	20.26 (12)	Si1—N1—C12—C13	−18.33 (17)
C15—Si1—N1—C12	109.40 (15)	Si1—O2—C13—O3	−169.03 (18)
C16—Si1—N1—C12	−65.89 (14)	Si1—O2—C13—C12	12.0 (2)
Si1—O1—C2—C1	31.3 (2)	N1—C12—C13—O3	−174.90 (19)
Si1—O1—C2—C3	−151.51 (14)	C14—C12—C13—O3	63.7 (2)
C11—C1—C2—O1	4.8 (2)	N1—C12—C13—O2	4.1 (2)
C9—C1—C2—O1	−178.98 (15)	C14—C12—C13—O2	−117.35 (18)
C11—C1—C2—C3	−172.31 (16)	O1—Si1—C16—C21	26.59 (14)
C9—C1—C2—C3	3.9 (3)	O2—Si1—C16—C21	−143.80 (14)
O1—C2—C3—C4	179.15 (18)	N1—Si1—C16—C21	−62.58 (16)
C1—C2—C3—C4	−3.6 (3)	C15—Si1—C16—C21	122.27 (16)
C2—C3—C4—C10	0.5 (3)	O1—Si1—C16—C17	−153.51 (13)
C10—C5—C6—C7	0.9 (4)	O2—Si1—C16—C17	36.10 (14)
C5—C6—C7—C8	−0.7 (4)	N1—Si1—C16—C17	117.32 (13)
C6—C7—C8—C9	−0.4 (4)	C15—Si1—C16—C17	−57.83 (18)
C7—C8—C9—C10	1.2 (3)	C21—C16—C17—C18	0.5 (2)
C7—C8—C9—C1	−177.1 (2)	Si1—C16—C17—C18	−179.42 (13)
C2—C1—C9—C8	177.35 (19)	C16—C17—C18—C19	1.7 (3)
C11—C1—C9—C8	−6.6 (3)	C17—C18—C19—C20	−1.9 (3)
C2—C1—C9—C10	−1.0 (2)	C18—C19—C20—C21	−0.1 (3)
C11—C1—C9—C10	175.05 (16)	C19—C20—C21—C16	2.3 (3)
C6—C5—C10—C9	−0.1 (3)	C17—C16—C21—C20	−2.5 (2)
C6—C5—C10—C4	179.5 (2)	Si1—C16—C21—C20	177.42 (13)

### (RS)-Methyl{N-[(2-oxidonaphthalen-1-yl)methylidene]alaninato}phenylsilicon chloroform hemisolvate (3) Hydrogen-bond geometry (Å, º)

*Cg*5 is defined as the centre of gravity of the ring with C16-C21.

**Table:**

*D*—H···*A*	*D*—H	H···*A*	*D*···*A*	*D*—H···*A*
C3—H3···O1^i^	0.95	2.62	3.562 (2)	171
C8—H8···O3^ii^	0.95	2.64	3.530 (3)	155
C11—H11···O3^ii^	0.95	2.34	3.278 (2)	169
C22—H22···O2^iii^	1.00	2.55	3.534 (5)	169
C4—H4···*Cg*5^i^	0.95	2.67	3.506 (2)	147

Symmetry codes: (i) −*x*+3/2, −*y*+1/2, −*z*+1/2; (ii) *x*+1/2, −*y*, *z*; (iii) *x*−1/2, −*y*, *z*.
